# Supplementary material for: Evolutionary and functional impact of common polymorphic inversions in the human genome
Source: Nat Commun. 2019 Sep 17;10:4222. doi: 10.1038/s41467-019-12173-x (PMC6748972; doi:10.1038/s41467-019-12173-x)
Supplement: Supplementary file 1 — Supplementary Information [file 41467_2019_12173_MOESM1_ESM.pdf]

Supplementary Information

Evolutionary and functional impact of common polymorphic inversions in the human genome

Carla Giner-Delgado[1,2 †], Sergi Villatoro[1 †], Jon Lerga-Jaso[1 †], Magdalena Gayà-Vidal[1,3], Meritxell Oliva[1], David Castellano[1], Lorena Pantano[1], Bárbara D. Bitarello[4], David Izquierdo[1], Isaac Noguera[1], Iñigo Olalde[5], Alejandra Delprat[1], Antoine Blancher[6,7], Carles Lalueza-Fox[5], Tõnu Esko[8], Paul F. O'Reilly[9], Aida M. Andrés[4,10], Luca Ferretti[11], Marta Puig[1], Mario Cáceres[1,12] *

[1] Institut de Biotecnologia i de Biomedicina, Universitat Autònoma de Barcelona, Bellaterra, Barcelona, 08193, Spain.

[2] Departament de Genètica i de Microbiologia, Universitat Autònoma de Barcelona, Bellaterra, Barcelona, 08193, Spain.

[3] CIBIO/InBIO Research Center in Biodiversity and Genetic Resources, Universidade do Porto, Vairão, Distrito do Porto, 4485-661, Portugal.

[4] Department of Evolutionary Genetics, Max Planck Institute for Evolutionary Anthropology, Leipzig, Saxony, 04103, Germany.

[5] Institute of Evolutionary Biology, CSIC-Universitat Pompeu Fabra, Barcelona, 08003, Spain.

[6] Laboratoire d'immunologie, CHU de Toulouse, IFB Hôpital Purpan, 31059, Toulouse, France.

[7] Centre de Physiopathologie Toulouse-Purpan (CPTP), Université de Toulouse, Centre National de la Recherche Scientifique (CNRS), Institut National de la Santé et de la Recherche Médicale (Inserm), Université Paul Sabatier (UPS), 31024, Toulouse, France.

[8] Estonian Genome Center, University of Tartu, Tartu, 51010, Estonia.

[9] Social, Genetic, and Developmental Psychiatry, King's College London, London, SE5 8AF, United Kingdom.

[10] UCL Genetics Institute, Department of Genetics, Evolution and Environment, University College London, London, WC1E 6BT, United Kingdom.

[11] Big Data Institute, Li Ka Shing Centre for Health Information and Discovery, University of Oxford, Oxford, OX3 7LF, United Kingdom.

[12] ICREA, Barcelona, 08010, Spain.

† These authors contributed equally to this work.

* Corresponding author.

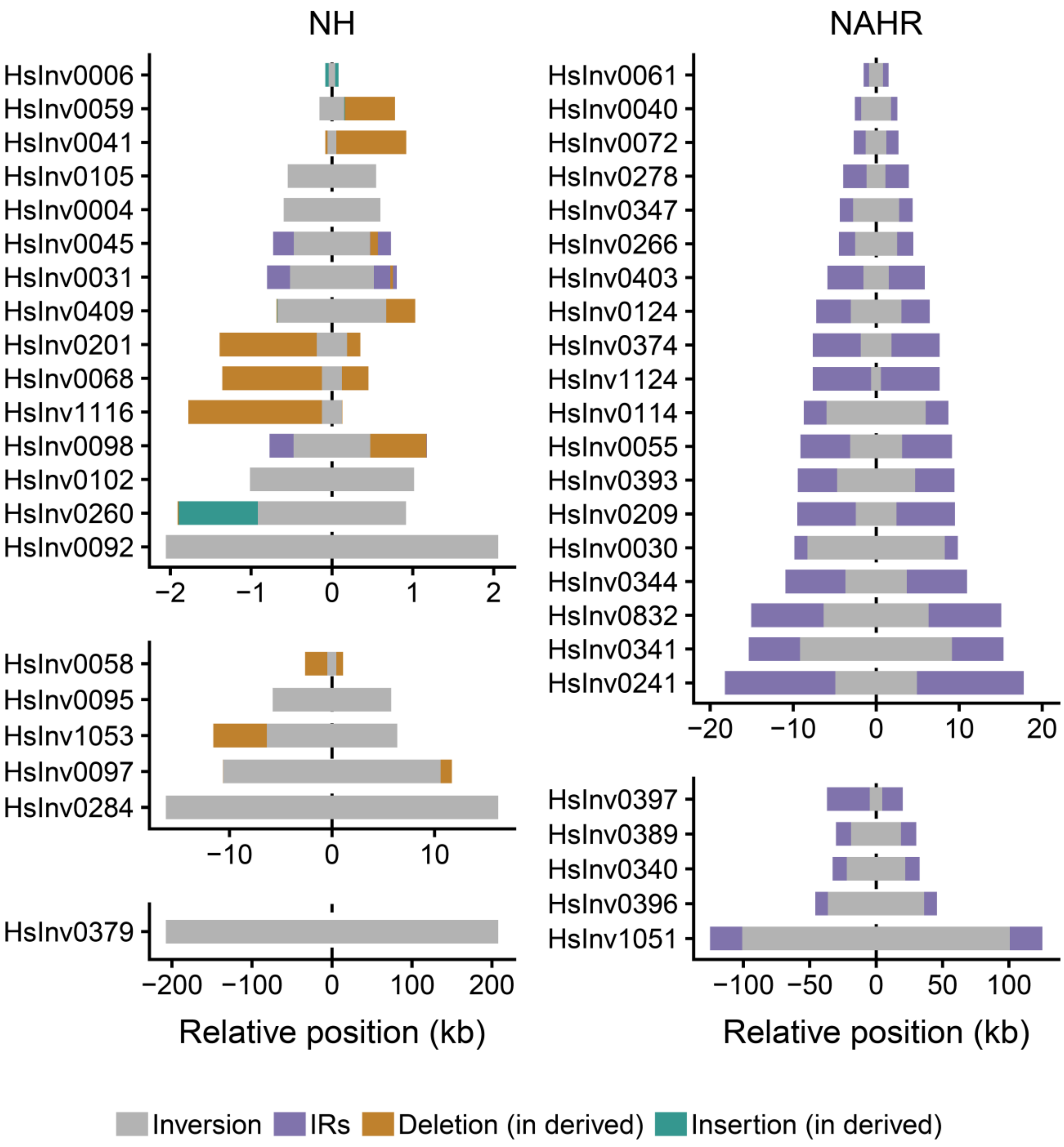

Supplementary Fig. 1. Size and breakpoint complexity of the 45 studied inversions. The graphs illustrate the main characteristics of inversions created by non-homologous mechanisms (NH) or non-allelic homologous recombination (NAHR), with the inverted region represented as a gray bar and flanking inverted repeats (IRs) or other structural changes in different colors. In NH inversions, deletions are sequences present in the original orientation that are eliminated in the derived orientation, and insertions are sequences gained. Three of these inversions (HsInv0031, HsInv0045 and HsInv0098) have also short low-identity IRs (249-297 bp, 83.2-86.2% identity) in the ancestral orientation that are partially deleted in the derived orientation. Source data are provided as a Source Data file.

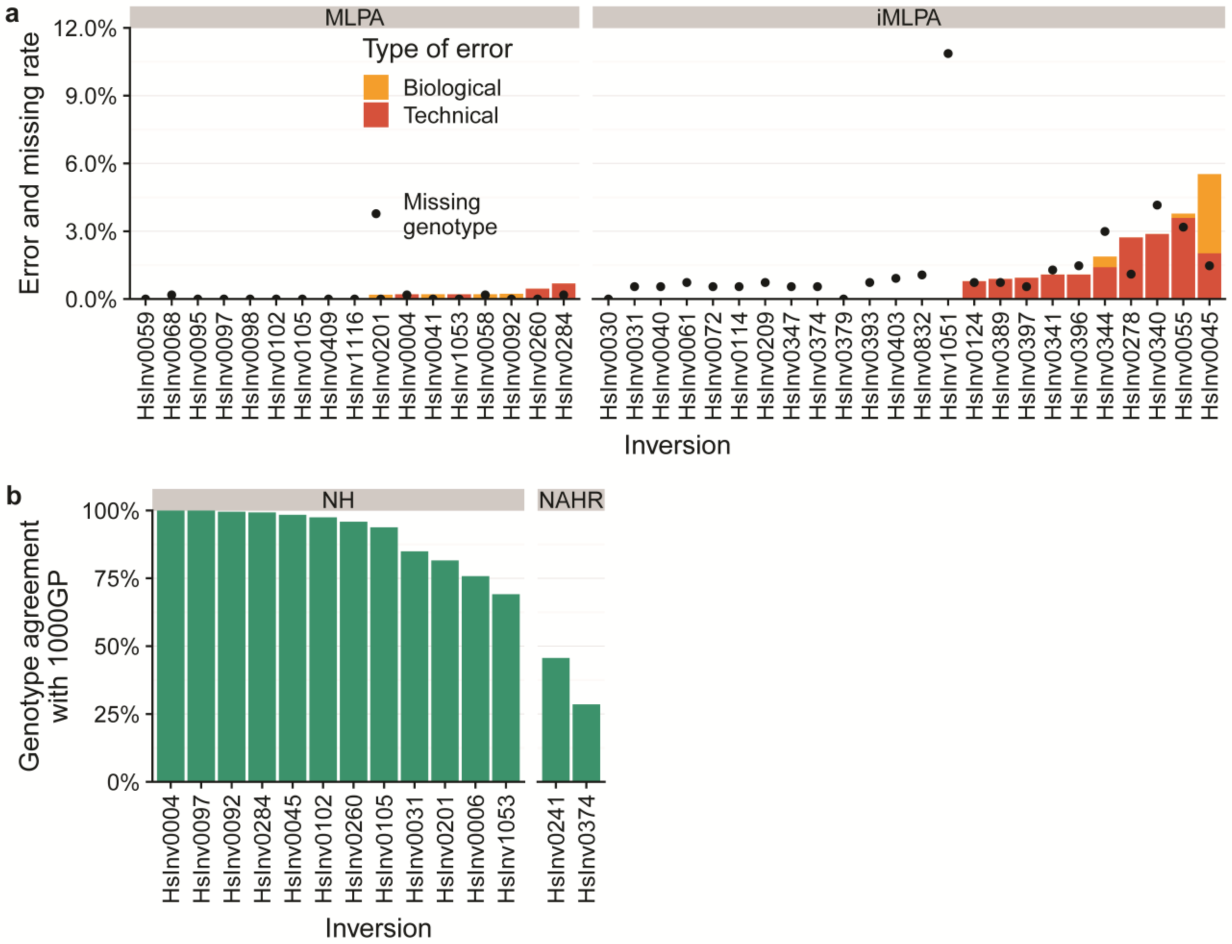

Supplementary Fig. 2. Inversion genotype accuracy by PCR-based validation and published data. **a.** Genotyping performance of MLPA and iMLPA assays. Inversion genotypes from MLPA/iMLPA were compared with those obtained from PCR or iPCR, plus those imputed from perfect tag SNPs in 1000GP Ph3 data. Genotyping success rate was 99.96% for MLPA and 98.56% for iMLPA. The lower success in iMLPA was due to a lower self-ligation efficiency of large restriction DNA fragments compared to shorter ones (as in the case of HsInv1051), which reduces the amount of specific probe target region and results in smaller amplification peaks, and to problems in specific samples (with one third of missing genotypes accumulating in just three samples). Biological errors correspond to known problems due to restriction site polymorphisms in a few specific inversions or DNA contamination, while technical errors do not have a clear cause and appear to be mainly due to problems in MLPA probe amplification in certain inversions. **b.** Genotype agreement between the 14 inversions in common with the 1000GP structural variant release[1] according to the InvFEST database[2] for the 434 samples shared in both datasets. Of the genotypes that differ between studies, 99.1% are due to 1000GP incorrectly assigning the reference genome orientation to one of the alleles, whereas according to our experiments it should be the alternative, which leads to underestimating the frequency of the inversion. Also, with a few exceptions, 1000GP error rates tend to be much higher in inversions flanked by indels or inverted repeats than in those with clean breakpoints. Source data are provided as a Source Data file.

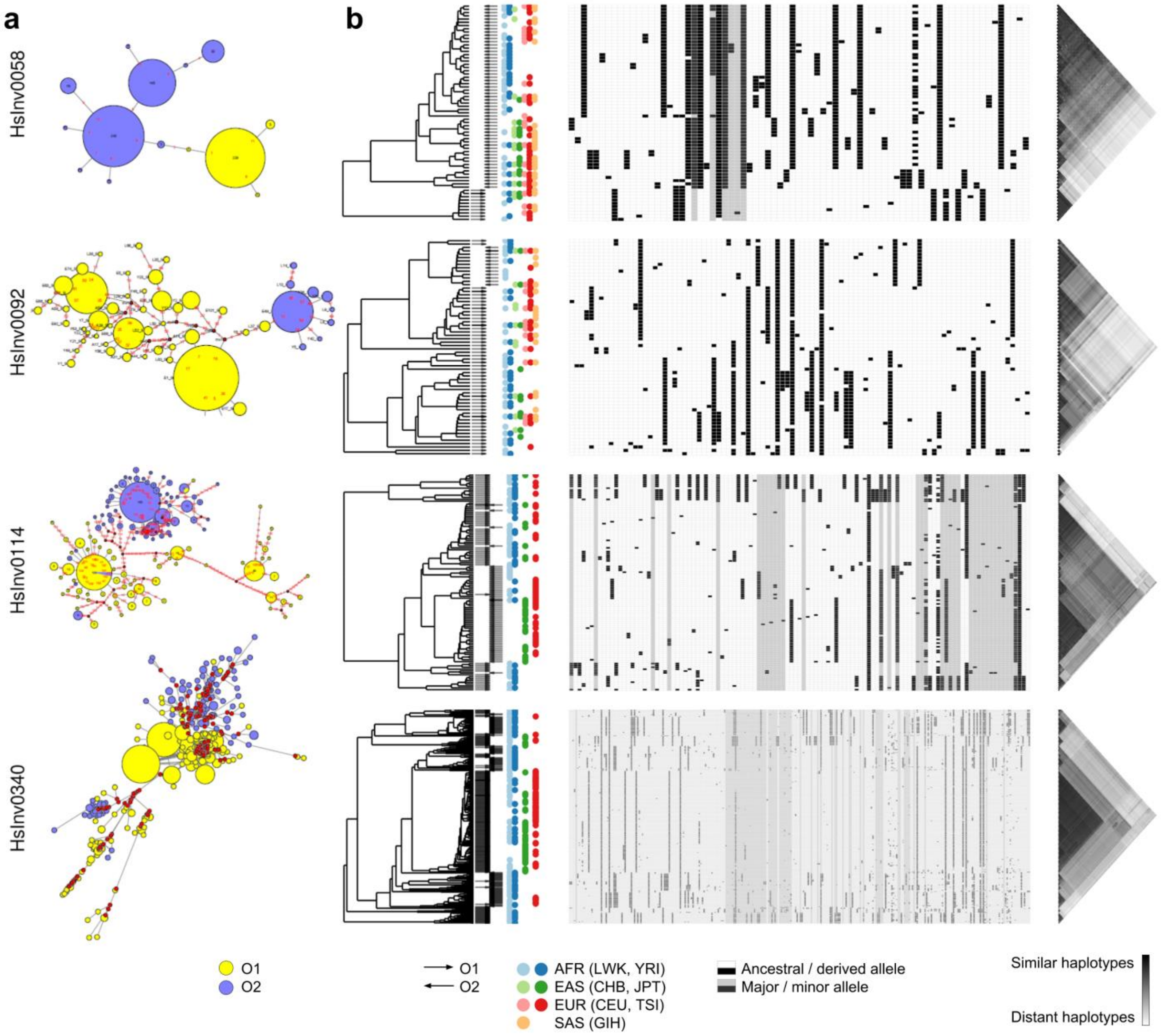

Supplementary Fig. 3. Summary of haplotype relationships for different inversions. a. Representative median-joining networks from 1000GP Ph1 haplotypes obtained with PHASE 2.1[3]. Each circle represents a haplotype, whose size is proportional to the number of chromosomes carrying that particular haplotype. Small red points are hypothetical haplotypes not found in the individuals analyzed, and the length of the branch connecting two haplotypes is proportional to the number of changes between them. **b.** Integrated haplotype plots (iHPlots) for the same four inversions. For unique inversions (HsInv0058 and HsInv0092), the haplotypes correspond to those from 1000GP Ph3 with the extended flanking region whenever possible, whereas for recurrent inversions (HsInv0114 and HsInv0340), the haplotypes are those obtained with PHASE 2.1[3] from 1000GP Ph1 data including only the inverted region. *O1* and *O2* haplotypes of unique inversions can be clearly separated (*e.g.* HsInv0058), probably corresponding to old inversions that had time to diverge, or those with the derived orientation can be clustered together with haplotypes carrying the other orientation (*e.g.* HsInv0092), likely representing more recent or small inversions with few informative positions and little differentiation.

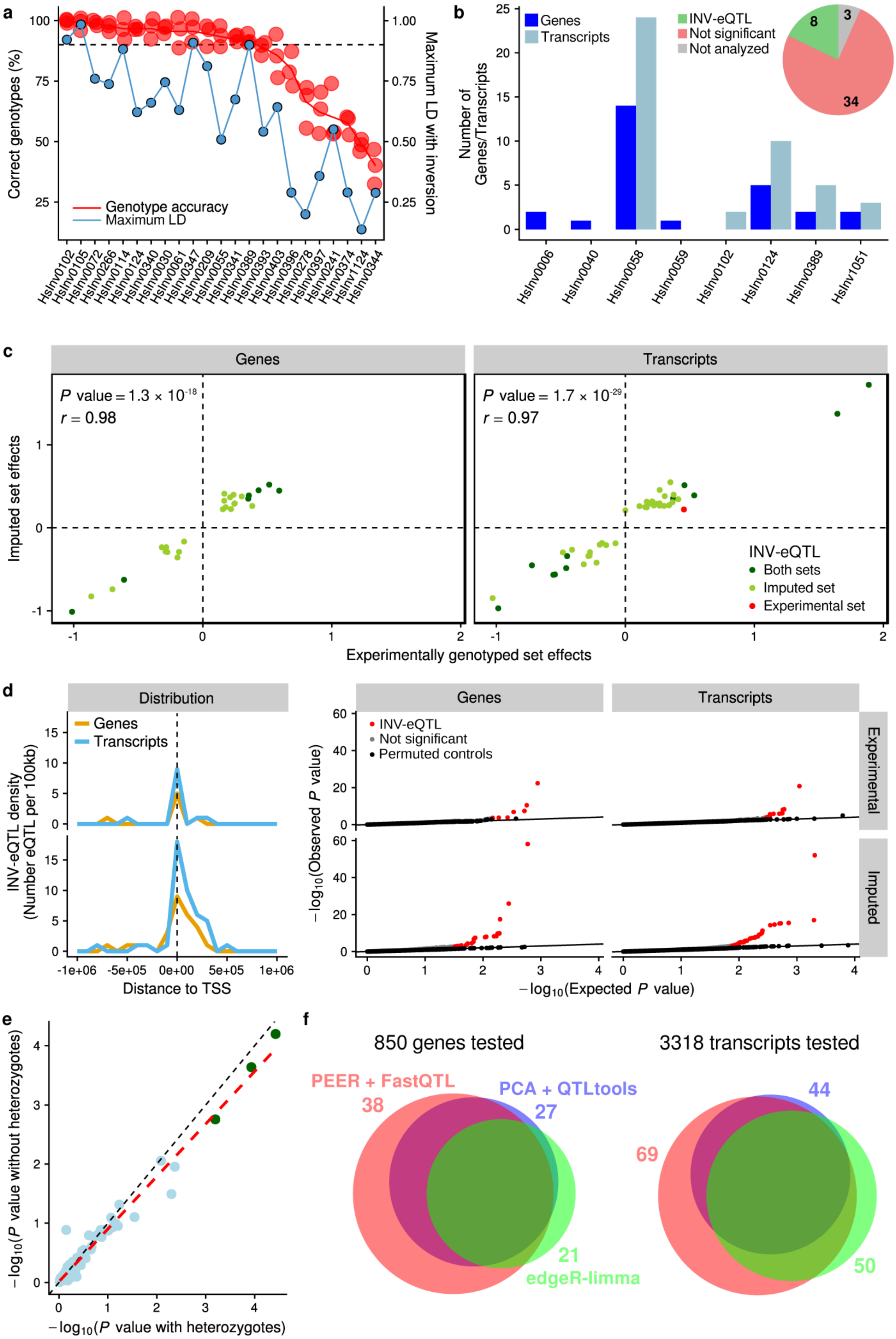
a
Correct genotypes (%)
Maximum LD with inversion
Genotype accuracy
Maximum LD
HsInv0102
HsInv0105
HsInv0072
HsInv0266
HsInv0114
HsInv0124
HsInv0340
HsInv0030
HsInv0061
HsInv0347
HsInv0209
HsInv0055
HsInv0341
HsInv0389
HsInv0393
HsInv0403
HsInv0396
HsInv0278
HsInv0397
HsInv0241
HsInv0374
HsInv1124
HsInv0344
b
Number of Genes/Transcripts
Genes
Transcripts
INV-eQTL
Not significant
Not analyzed
8
3
34
HsInv0006
HsInv0040
HsInv0058
HsInv0059
HsInv0102
HsInv0124
HsInv0389
HsInv1051
c
Genes
Transcripts
P value = 1.3 × 10-18
r = 0.98
P value = 1.7 × 10-29
r = 0.97
Imputed set effects
Experimentally genotyped set effects
INV-eQTL
Both sets
Imputed set
Experimental set
d
Distribution
Genes
Transcripts
INV-eQTL density (Number eQTL per 100kb)
Distance to TSS
Genes
Transcripts
INV-eQTL
Not significant
Permuted controls
Experimental
Imputed
−log10(Observed P value)
−log10(Expected P value)
e
−log10(P value without heterozygotes)
−log10(P value with heterozygotes)
f
850 genes tested
3318 transcripts tested
PEER + FastQTL
38
PCA + QTLtools
27
21
edgeR-limma
69
44
50

Supplementary Fig. 4. Summary of inversion gene-expression analysis in lymphoblastoid cell lines (LCL). a. Genotype imputation accuracy of 23 autosomal and chr. X inversions without perfect tag SNPs (r^2 = 1) based on all 1000GP Ph3 variants (including both SNPs and structural variants). Imputation was performed with IMPUTE v2.3.2[4] adapted to unphased reference genotypes, due to the difficulty of phasing correctly recurrent inversions, using a region of 1.5 Mb at each side of the inversion and an effective population size of 20,000 (15,000 in chr. X). Genotypes were called with a posterior probability higher than 0.7, and were classified as missing otherwise. Imputation accuracy was checked by removing three random sets of 30 individuals from European (CEU, TSI) and YRI populations from the reference panel of 173 GEUVADIS individuals with known inversion genotypes, which were subsequently imputed under the same criteria. The red line represents the mean percentage of right calls in the three test samples (red dots) and 14 inversions with >90% imputation accuracy (dashed line) were used for gene-expression analysis in other GEUVADIS individuals. Maximum LD between inversions and surrounding genomic variants (blue line) was lower for inversions with worse imputation accuracy. In HsInv0102, which does not have SNPs in high LD, its imputation is based on the 1000GP genotypes for the inversion itself. **b.** Pie chart and graph summarizing the effects in *cis* of the 45 inversions on LCL expression variation and the number of genes or transcripts affected. Results represented correspond to those from the experimentally-genotyped set (173 individuals) for the 9 inversions that could not be imputed and the extended imputed set (445 individuals) for the 33 imputed inversions. **c.** Comparison of effect sizes on genes and transcripts from inversion *cis*-eQTLs (INV-eQTLs) identified in the experimentally-genotyped and imputed sets in LCLs, showing concordant results (dark green, replicated in both sets; light green, specific of the imputed set; and red, specific of the experimentally-genotyped set). **d.** *Cis*-eQTL analysis of inversions in LCL expression data. Left: Distribution of INV-eQTLs with respect to the transcription start site (TSS) of the affected genes and transcripts. Inversions tend to locate closer (<100 kb) to genes or transcripts affected compared to all association tests performed both for the experimental (top, Fisher test P = 0.013 and P = 0.0005, respectively) and imputed data sets (bottom, Fisher test P = 0.018 and $P = 3 \times 10^{-6}$, respectively). Right: Quantile-quantile plot of associations between inversions and gene or transcript expression for the experimentally-genotyped and the imputed sets: red dots, significant INV-eQTLs (FDR < 0.05); grey dots, not significant associations; and black dots, negative controls obtained by permuting sample labels from the inversion genotype matrix relative to covariates and expression levels, which follow the expected P value distribution assuming no-association. **e.** Correlation of gene eQTL analysis P values for inversions located in chr. X with and without heterozygous females (to eliminate the effect of the random inactivation of one copy of this chromosome). Significant associations (FDR < 0.05) in both analyses are indicated as green dots, and the similarity between the observed and perfect 1:1 correlation (red and black dashed lines, respectively), with slightly lower eQTL P values when including all samples, suggests that the consequences of silencing the chr. X with or without the inversion get averaged across all cells. **f.** Results of inversion effects in gene and transcript expression when using different approaches: “PCA+QTLtools”, which corresponds to the pipeline used in this work[5] (blue); “PEER+FastQTL”, which corresponds to the pipeline used in the GTEx Project[6] (red); and “edgeR-limma”[7,8] (green). Numbers indicate the significant inversion-gene or inversion-transcript pairs with each analysis method. Venn diagram was done with BioVenn[9]. Findings using the different pipelines were highly coincident, although a larger number of significant genes/transcripts were estimated by the GTEx pipeline, indicating that our chosen method based in PCA and QTLtools is more conservative. Source data are provided as a Source Data file.

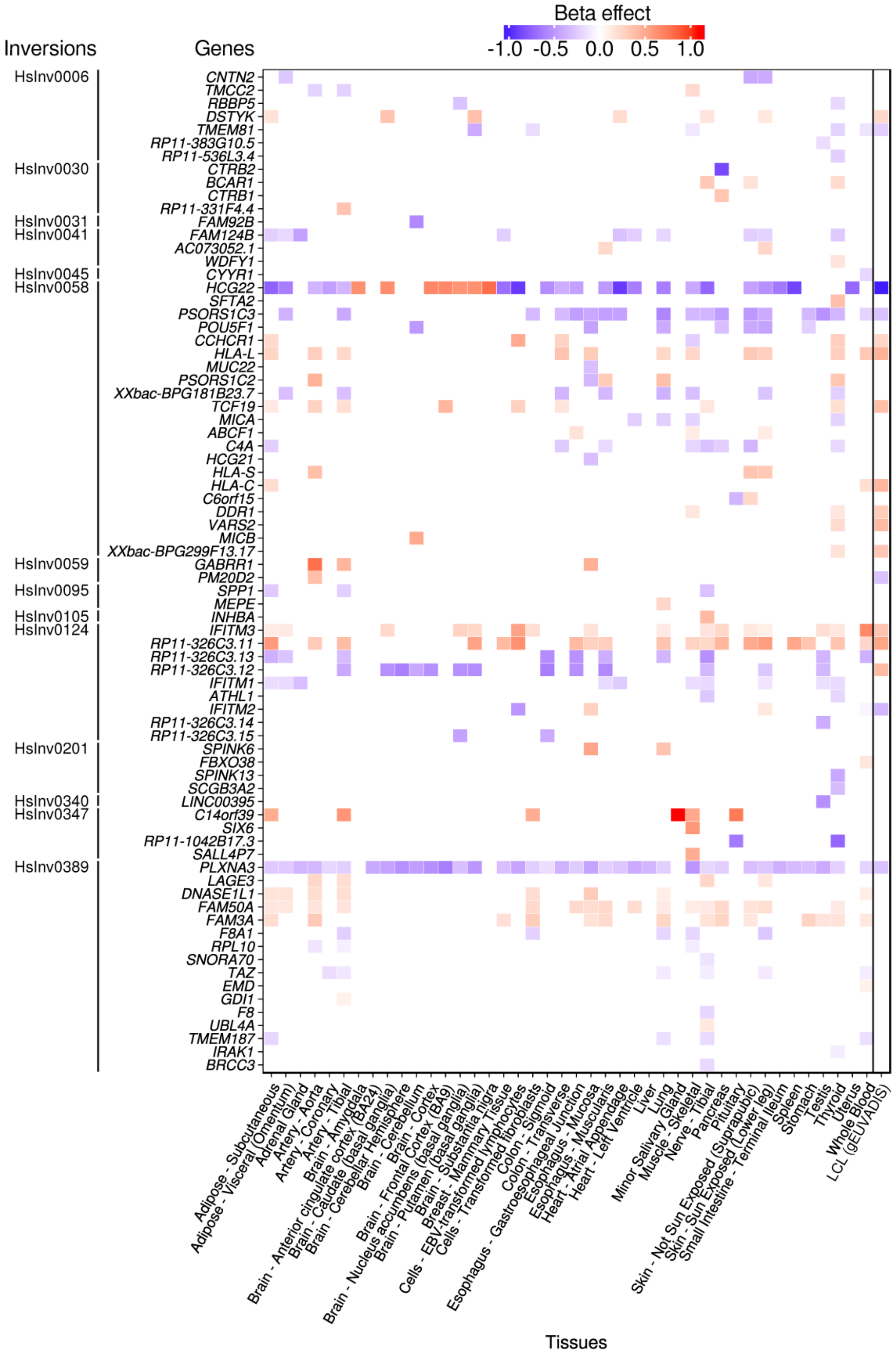

Supplementary Fig. 5. Summary of inversion effects on GTEX gene-expression data. Inversion effects were estimated through variants in high LD (r^2 ≥ 0.8), or moderate LD (r^2 ≥ 0.6) for recurrent inversions, reported as eQTLs in GTEx Analysis Release v7 (Supplementary Data 9). The direction and strength of the beta effect of the eQTL is indicated in different color, with blue and red representing respectively lower and higher expression associated to the *O2* orientation of the inversion. Inversion eQTLs also identified in the LCL analysis from the GEUVADIS data are represented in the last column. Source data are provided as a Source Data file.

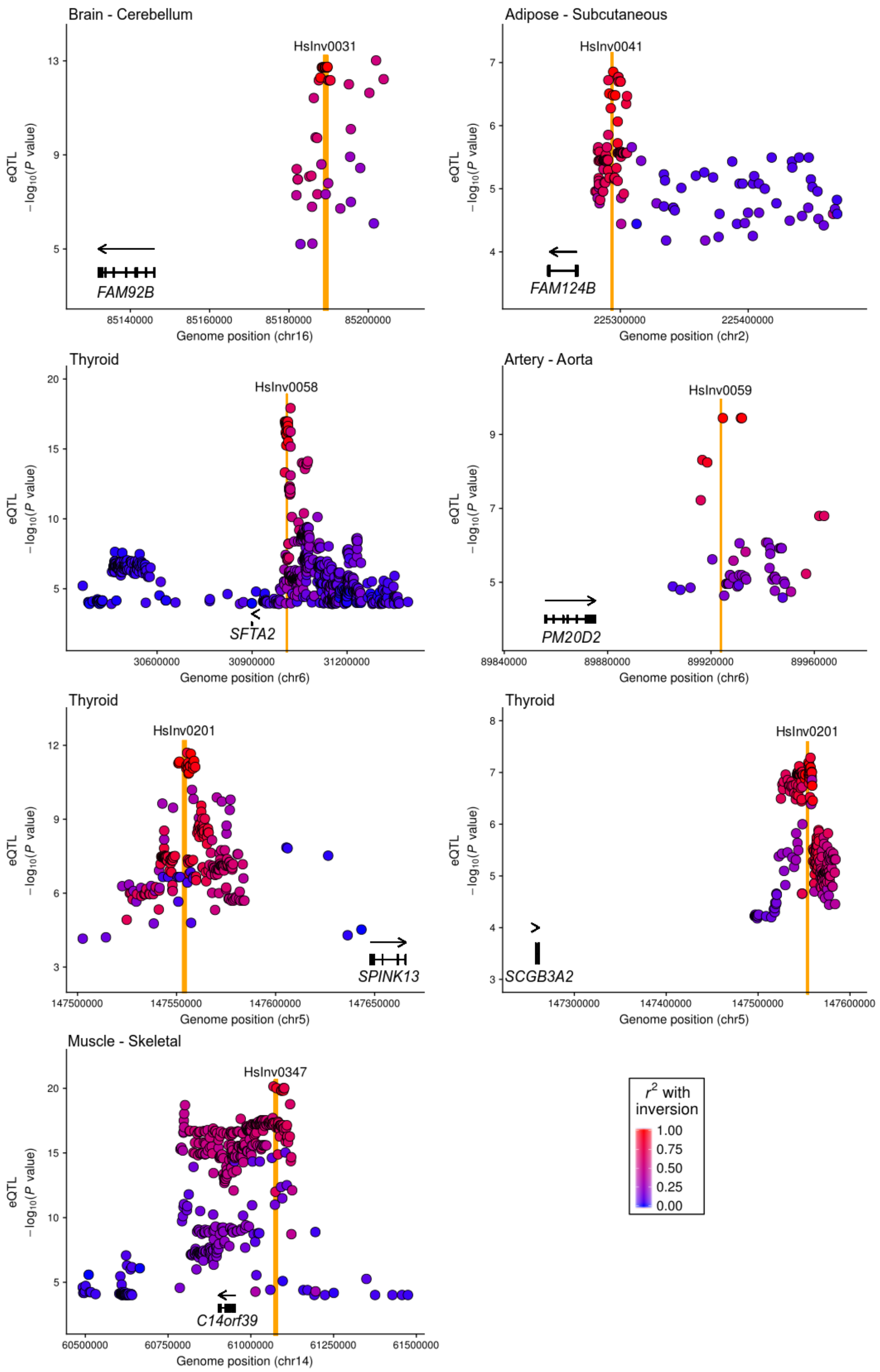

Supplementary Fig. 6. Examples of potential expression effects of six inversions in different tissues. Manhattan plots of logarithm-transformed linear regression t-test *P* values for *cis*-eQTLs associations from the GTEx project in which an inversion shows the highest LD ($r^2 \geq 0.9$) with the two first lead markers in the corresponding tissue. The orange bar pinpoints the inversion position and its LD to each variant is represented in different colors. The affected genes are shown in black and arrowheads indicate the direction of transcription.

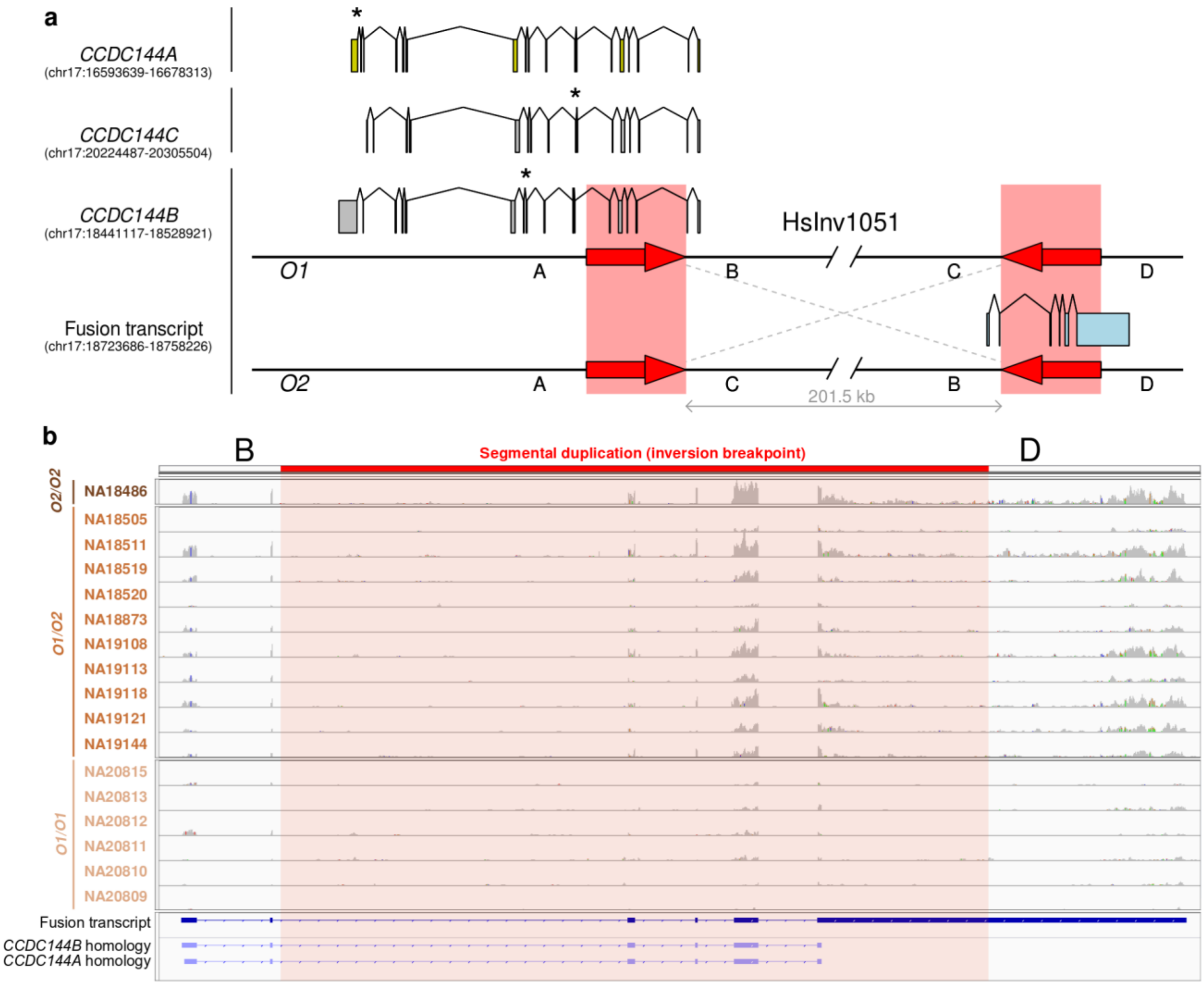

Supplementary Fig. 7. Representation of the fusion transcript created by HsInv1051. a. Diagram of *CCDC144B* gene disruption by inversion HsInv1051 and the novel fusion transcript created by including additional 3’ sequences from region D (light blue), with the segmental duplications at the inversion breakpoints represented as red arrows. *CCDC144B* is part of a family with two other members, *CCDC144A* and *CCDC144C*, that have ~99% identity and very similar exon-intron structure (shown on top). Nevertheless, whereas *CCDC144A* encodes a 1,427-amino acid protein, *CCDC144B* and *CCDC144C* have different frameshift mutations that reduce their coding capacity to 725 and 646 amino acids, respectively (with stop codons shown by asterisks). *CCDC144B* premature stop codon is not included in the fusion transcript from the inverted allele. **b.** RNA-Seq profiles from GEUVADIS LCL reads mapped to the inversion BD breakpoint, which was created by reversing *in silico* the sequence between the HsInv1051 breakpoints in the human reference genome (hg19). Reads were remapped to this construct using STAR 2-pass[10] to improve the accuracy of alignments, revealing a novel fusion transcript expressed only in *O1/O2* heterozygotes and at higher levels in *O2/O2* homozygotes. The chimeric transcript structure is shown below, after its precise reconstruction with Cufflinks default parameters[11] by merging all reads from these samples around the breakpoint region. In addition, its homology with the first six exons of *CCDC144B* and *CCDC144A* is also shown. RNA-seq profiles were visualized on Integrative Genomics Viewer[12].

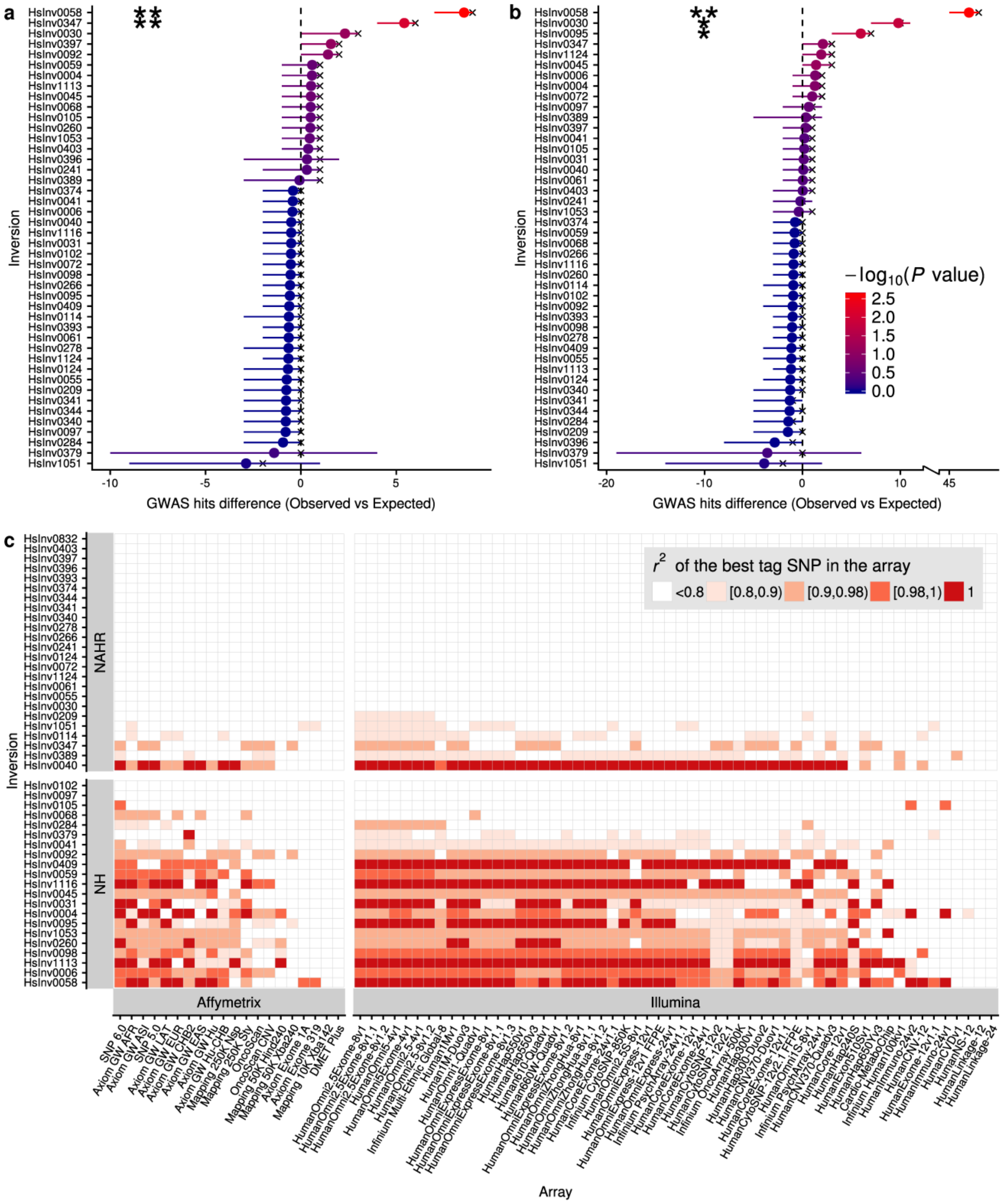

Supplementary Fig. 8. Potential phenotypic effects of inversions from GWAS data. a-b. Enrichment of GWAS signals around 44 autosomal and chr. X inversions (inverted region ± 20 kb) in the GWAS Catalog (**a**) and GWASdb (**b**) databases. Error bars show the 0-0.95 confidence interval of the difference in the observed number of GWAS hits compared with a background model from 1,000 random genomic regions for each inversion, together with the mean (filled circle) and the median (cross) of the differences. The color indicates the one-tailed empirical test *P* value of the enrichment according to the scale shown. HsInv0058 showed significant enrichment of GWAS hits in both datasets, whereas HsInv0030 and HsInv0347 showed similar trends in both datasets and significant differences from the expected number in at least one. *, $P < 0.05$; **, $P < 0.01$. **c.** Coverage of SNPs associated with inversions in 76 commonly-used genotyping arrays by checking the presence of inversion global tag SNPs ($r^2 \geq 0.8$) in the arrays through the LDLink web portal[13]. LD with the inversion of the best global tag SNP in each array is indicated in different colors, showing that for the great majority of NAHR inversions

and several of the NH inversions there are not tag SNPs or they are not present in the array (represented as white squares). The best performing arrays assessed, HumanOmni5-4v1 and HumanOmni5Exome-4v1 (Illumina), could detect up to 23 inversions (51%), with only 7 being represented by perfect global tag SNPs (r^2 = 1), and 16 by variants with lower LD. Source data are provided as a Source Data file.

Supplementary Table 1. Frequencies of the 45 inversions in seven human populations. The derived allele frequency (DAF) is shown whenever the ancestral orientation is known, and the frequency of the minor allele considering the seven populations together (MAF) is indicated otherwise. The total number of genotyped individuals, as well as those unrelated (Unrel) and included in either the 1000 Genome Project Phase 1 (Ph1) or Phase 3 (Ph3), are also indicated for each population and population group at the bottom. Inversion frequency was estimated from the 480 unrelated individuals of the seven known populations, although for some analyses only the 434 individuals in common with 1000GP Ph3 were used. Deviation from Hardy-Weinberg equilibrium was calculated with Plink --hardy option[14] and for all populations and inversions an exact test $P > 0.01$ was obtained. Populations are: Luhya in Webuye, Kenya (LWK); Yoruba in Ibadan, Nigeria (YRI); Utah residents (CEPH) with Northern and Western European ancestry (CEU); Toscani in Italia (TSI); Gujarati Indians in Houston, Texas, USA (GIH); Han Chinese in Beijing, China (CHB); and Japanese in Tokyo, Japan (JPT). Population groups are: African ancestry (AFR); European ancestry (EUR); South-Asian ancestry (SAS); and East-Asian ancestry (EAS).

Inversion	Allele	Population or population group											Total
		LWK	YRI	AFR	CEU	TSI	EUR	GIH	SAS	CHB	JPT	EAS	
**Derived allele frequency (DAF)**													
**HsInv0004**	***O1***	0.981	0.993	**0.987**	0.800	0.828	**0.817**	0.837	**0.837**	0.856	0.889	**0.872**	**0.884**
**HsInv0006**	***O1***	0.931	0.943	**0.937**	0.408	0.356	**0.377**	0.511	**0.511**	0.456	0.400	**0.428**	**0.587**
**HsInv0030**	***O1***	0.012	0.021	**0.017**	0.158	0.156	**0.157**	0.062	**0.062**	0	0	**0**	**0.066**
**HsInv0031**	***O1***	0.481	0.371	**0.430**	0.317	0.281	**0.295**	0.399	**0.399**	0.433	0.432	**0.433**	**0.383**
**HsInv0040**	***O1***	0.228	0.279	**0.252**	0.208	0.382	**0.312**	0.191	**0.191**	0.100	0.044	**0.072**	**0.225**
**HsInv0041**	***O2***	0.747	0.629	**0.692**	0.442	0.428	**0.433**	0.371	**0.371**	0.411	0.422	**0.417**	**0.500**
**HsInv0045**	***O2***	0.463	0.636	**0.543**	0.450	0.561	**0.517**	0.393	**0.393**	0.611	0.544	**0.578**	**0.514**
**HsInv0058**	***O1***	0.222	0.384	**0.297**	0.383	0.350	**0.363**	0.258	**0.258**	0.456	0.456	**0.456**	**0.340**
**HsInv0059**	***O1***	0.093	0.093	**0.093**	0.183	0.161	**0.170**	0.180	**0.180**	0.722	0.678	**0.700**	**0.247**
**HsInv0061**	***O1***	0	0	**0**	0.025	0.034	**0.030**	0.006	**0.006**	0	0.022	**0.011**	**0.013**
**HsInv0068**	***O1***	0.099	0.116	**0.107**	0.225	0.233	**0.230**	0.112	**0.112**	0	0	**0**	**0.126**
**HsInv0092**	***O2***	0.265	0.350	**0.305**	0.067	0.089	**0.080**	0.129	**0.129**	0.078	0.089	**0.083**	**0.160**
**HsInv0095**	***O1***	0.173	0.121	**0.149**	0.325	0.289	**0.303**	0.157	**0.157**	0.256	0.244	**0.250**	**0.218**
**HsInv0097**	***O2***	0.019	0.014	**0.017**	0	0	**0**	0	**0**	0	0	**0**	**0.005**
**HsInv0098**	***O2***	0.346	0.307	**0.328**	0.117	0.111	**0.113**	0.096	**0.096**	0.078	0.078	**0.078**	**0.171**
**HsInv0102**	***O2***	0.272	0.350	**0.308**	0.133	0.156	**0.147**	0.202	**0.202**	0.033	0.044	**0.039**	**0.188**
**HsInv0105**	***O1***	0.469	0.450	**0.460**	0.517	0.639	**0.590**	0.618	**0.618**	0.256	0.211	**0.233**	**0.488**
**HsInv0114**	***O1***	0.800	0.843	**0.820**	0.375	0.354	**0.362**	0.348	**0.348**	0.144	0.156	**0.150**	**0.463**
**HsInv0201**	***O2***	0.611	0.664	**0.636**	0.533	0.644	**0.600**	0.506	**0.506**	0.489	0.367	**0.428**	**0.561**
**HsInv0209**	***O2***	0.184	0.271	**0.225**	0.017	0.084	**0.057**	0	**0**	0.022	0.011	**0.017**	**0.091**
**HsInv0260**	***O2***	0.204	0.129	**0.169**	0.125	0.083	**0.100**	0.174	**0.174**	0.289	0.422	**0.356**	**0.183**
**HsInv0266**	***O2***	0.269	0.271	**0.270**	0.208	0.178	**0.190**	0.500	**0.500**	0.250	0.289	**0.270**	**0.288**
**HsInv0278**	***O1***	0.608	0.543	**0.577**	0.898	0.909	**0.905**	0.843	**0.843**	0.733	0.656	**0.694**	**0.751**
**HsInv0284**	***O2***	0.105	0.101	**0.103**	0	0	**0**	0	**0**	0	0	**0**	**0.032**
**HsInv0379**	***O2***	0	0	**0**	0	0	**0**	0	**0**	0.022	0.033	**0.028**	**0.005**
**HsInv0409**	***O1***	0.385	0.359	**0.373**	0.478	0.630	**0.569**	0.455	**0.455**	0.662	0.776	**0.719**	**0.515**
**HsInv1051**	***O2***	0.029	0.080	**0.055**	0	0	**0**	0	**0**	0	0	**0**	**0.018**
**HsInv1053**	***O2***	0.247	0.236	**0.242**	0.692	0.600	**0.637**	0.702	**0.702**	0.722	0.689	**0.706**	**0.538**
**HsInv1116**	***O2***	0.722	0.843	**0.778**	0.750	0.711	**0.727**	0.938	**0.938**	1	1	**1**	**0.833**

(Continued in next page)

Inversion	Allele	Population or population group											Total
		LWK	YRI	AFR	CEU	TSI	EUR	GIH	SAS	CHB	JPT	EAS	
**Minor allele frequency (MAF)**													
**HsInv0055**	***O1***	0.605	0.557	**0.583**	0.217	0.225	**0.221**	0.247	**0.247**	0.156	0.122	**0.139**	**0.325**
**HsInv0072**	***O1***	0.050	0.078	**0.063**	0.011	0.008	**0.009**	0.007	**0.007**	0	0	**0**	**0.024**
**HsInv0124**	***O1***	0.146	0.121	**0.134**	0.608	0.551	**0.574**	0.281	**0.281**	0.022	0.056	**0.039**	**0.281**
**HsInv0241**	***O2***	0.582	0.597	**0.589**	0.167	0.184	**0.177**	0.301	**0.301**	0.405	0.378	**0.391**	**0.370**
**HsInv0340**	***O2***	0.513	0.507	**0.510**	0.008	0.034	**0.024**	0.011	**0.011**	0	0	**0**	**0.167**
**HsInv0341**	***O2***	0.152	0.257	**0.201**	0.025	0.022	**0.024**	0.017	**0.017**	0	0.023	**0.011**	**0.076**
**HsInv0344**	***O2***	0.487	0.493	**0.490**	0.542	0.483	**0.507**	0.354	**0.354**	0.411	0.278	**0.344**	**0.442**
**HsInv0347**	***O2***	0.234	0.286	**0.258**	0.092	0.118	**0.107**	0.303	**0.303**	0.133	0.122	**0.128**	**0.195**
**HsInv0374**	***O2***	0.392	0.243	**0.322**	0.467	0.461	**0.463**	0.601	**0.601**	0.533	0.711	**0.622**	**0.475**
**HsInv0389**	***O2***	0.958	1	**0.978**	0.178	0.173	**0.175**	0.478	**0.478**	0.221	0.313	**0.267**	**0.499**
**HsInv0393**	***O2***	0.317	0.330	**0.323**	0.367	0.391	**0.381**	0.657	**0.657**	0.647	0.746	**0.696**	**0.474**
**HsInv0396**	***O2***	0.263	0.417	**0.335**	0.159	0.144	**0.150**	0.209	**0.209**	0.029	0.015	**0.023**	**0.195**
**HsInv0397**	***O2***	0.608	0.553	**0.583**	0.156	0.158	**0.157**	0.291	**0.291**	0.456	0.687	**0.570**	**0.393**
**HsInv0403**	***O2***	0.650	0.650	**0.650**	0.267	0.189	**0.221**	0.328	**0.328**	0.824	0.672	**0.748**	**0.475**
**HsInv0832**	***O2***	0.821	1	**0.908**	0	0	**0**	0.250	**0.250**	0	0.043	**0.022**	**0.339**
**HsInv1124**	***O2***	0.375	0.551	**0.456**	0.625	0.589	**0.603**	0.365	**0.365**	0.409	0.600	**0.506**	**0.495**
**Total indiv.**	-	90	100	**190**	90	90	**180**	90	**90**	45	45	**90**	**550**
**Unrel. indiv.**	-	81	70	**151**	60	90	**150**	89	**89**	45	45	**90**	**480**
**Ph1 indiv.**	-	87	48	**135**	35	90	**125**	0	**0**	41	39	**80**	**340**
**Ph3 indiv.**	-	75	58	**133**	45	89	**134**	82	**82**	40	45	**85**	**434**

Supplementary Table 2. Summary of the total number of analyzed, shared and fixed variants in human inversions from 1000 Genomes Project (1000GP) Phase3 and HapMap genotype data. For 1000GP data only accessible variants according to the strict criteria were used. As expected, most NH inversions have no shared variants between orientations within the inverted region. The only exceptions were HsInv1053, with a single shared SNP near the second breakpoint in 1000GP and two shared SNPs in HapMap, and HsInv0095, with shared variants only in HapMap. These variants tend to be grouped in certain positions and are likely the result of gene conversion, SNP genotyping errors or even independent mutations. In addition, there is considerable variation in the number of fixed variants between inversions, which is probably related to the recombination events outside the inverted region. Non-recombining flanking region was estimated according to the distribution of fixed and shared variants up to a maximum 20 kb from the breakpoints. NA, Not applicable.

	Inside				Inside + 200 kb		Non-recombining flanking region (kb)	
	Analyzed variants		Shared variants		Fixed variants			
Inversion	1000GP	HapMap	1000GP	HapMap	1000GP	HapMap	Upstream	Downstream
**Inversions generated by non-homologous mechanisms (NH)**								
**HsInv0004**	21	2	0	0	18	2	1.9	11.7
**HsInv0006**	0	0	NA	NA	4	0	3.1	0.1
**HsInv0031**	8	4	0	0	9	2	0.5	0
**HsInv0041**	0	1	NA	0	2	0	0	0.2
**HsInv0045**	4	1	0	0	1	0	0	1.3
**HsInv0058**	0	1	NA	0	8	2	0.9	2.6
**HsInv0059**	0	0	NA	NA	1	0	4.3	4.7
**HsInv0068**	0	1	NA	0	1	1	3	7.7
**HsInv0092**	56	1	0	0	1	0	2.8	3.2
**HsInv0095**	101	7	0	3	4	1	3.9	2.1
**HsInv0097**	247	18	0	0	17	0	20	20
**HsInv0098**	8	0	0	NA	8	0	10.9	0.4
**HsInv0102**	14	0	0	NA	0	0	0	0.6
**HsInv0105**	1	0	0	NA	0	1	0.5	20
**HsInv0201**	0	0	NA	NA	16	3	3.3	4.7
**HsInv0260**	12	1	0	0	3	0	19.8	1.8
**HsInv0284**	418	13	0	0	3	0	1.6	18.3
**HsInv0379**	3426	155	0	0	3	0	18.1	15.6
**HsInv0409**	0	0	NA	NA	1	1	0	0.3
**HsInv1053**	185	12	1	2	2	0	0.2	0.3
**HsInv1116**	0	0	NA	NA	22	3	7.7	2

(Continued in next page)

Inversion	Inside				Inside + 200 kb		Non-recombining flanking region (kb)	
	Analyzed variants		Shared variants		Fixed variants			
	1000GP	HapMap	1000GP	HapMap	1000GP	HapMap	Upstream	Downstream
**Inversions generated by non-allelic homologous recombination (NAHR)**								
**HsInv0030**	225	11	17	6	0	0	NA	NA
**HsInv0040**	33	0	0	NA	43	0	9.1	20
**HsInv0055**	21	2	5	2	0	0	NA	NA
**HsInv0061**	10	0	0	NA	0	0	NA	NA
**HsInv0072**	14	1	1	0	0	0	NA	NA
**HsInv0114**	141	12	10	4	0	0	NA	NA
**HsInv0124**	58	0	8	NA	0	0	NA	NA
**HsInv0209**	71	4	9	3	0	0	NA	NA
**HsInv0241**	46	3	18	3	0	0	NA	NA
**HsInv0266**	40	0	2	NA	0	0	NA	NA
**HsInv0278**	18	0	6	NA	0	0	NA	NA
**HsInv0340**	170	6	45	5	0	0	NA	NA
**HsInv0341**	196	11	33	11	0	0	NA	NA
**HsInv0344**	37	2	15	2	0	0	NA	NA
**HsInv0347**	44	2	13	1	0	0	NA	NA
**HsInv0374**	1	1	0	1	0	0	NA	NA
**HsInv0389**	247	7	39	7	0	0	NA	NA
**HsInv0393**	73	1	11	1	0	0	NA	NA
**HsInv0396**	399	14	75	14	0	0	NA	NA
**HsInv0397**	98	0	18	NA	0	0	NA	NA
**HsInv0403**	10	0	4	NA	0	0	NA	NA
**HsInv0832**	0	0	NA	NA	0	0	NA	NA
**HsInv1051**	67	30	0	0	1	0	NA	NA
**HsInv1124**	6	1	3	1	0	0	NA	NA

Supplementary Table 3. Summary of inversion mutational effects on gene sequences. Gene annotations are based on GENCODE Version 26 Comprehensive Gene Annotation Set, including gene isoforms with a Transcript Support Level of at least 3, single-exon genes not labelled as “problem”, and pseudogenes. Effects of inversions and associated indels at the breakpoints were classified conservatively in six different categories: (1) gene disruption, if there is at least one transcript that encompasses the complete area of one breakpoint; (2) exchange of genic sequences, if two genes of the same family overlap each inversion breakpoint and extend outside of them; (3) inversion of a gene/exon, if the entire gene/exon is situated within the inverted region; (4) inversion of part of an intron, if the inversion and breakpoints are contained inside an intron; (5) overlap of breakpoints with genes within IRs, if there are genes completely embedded within IRs at the inversion breakpoints; and (6) intergenic, if none of the above conditions are fulfilled. For genes overlapping inversion breakpoints within IRs, there could be a potential disruption or exchange of gene sequences, although it is difficult to determine its precise effect due to the high identity of the IRs.

Inversion	Effect	Protein-coding genes	Long non-coding RNAs	Pseudogenes	Other
**HsInv0006**	Inversion of part of intron	*DSTYK*			
**HsInv0030**	Exchange of genic sequences	*CTRB1, CTRB2*			
**HsInv0055**	Inversion of part of intron			*AC016561.1*	
**HsInv0059**	Inversion of part of intron	*GABRR1*			
**HsInv0061**	Inversion of part of intron		*RP1-60O19.1*		
**HsInv0098**	Inversion of part of intron	*ULK4*			
**HsInv0102**	Inversion or deletion of an exon	*RHOH* isoform			
**HsInv0105**	Inversion of part of intron	*SUGCT*			
**HsInv0124**	Gene disruption	*IFITM2* isoform			
	Inversion of whole gene	*IFITM1*			
	Breakpoints overlap genes within IRs		*RP11-326C3.7, RP11-326C3.11*		
**HsInv0201**	Inversion or deletion of an exon	*SPINK14*			
**HsInv0209**	Breakpoints overlap genes within IRs	*KRTAP5-10, KRTAP5-11, AP000867.1*		*AP000867.14, KRTAP5-14P*	
**HsInv0241**	Breakpoints overlap genes within IRs	*AQP12A, AQP12B*			
	Inversion of whole gene	*AC011298.1*	*AC011298.2*		
**HsInv0278**	Inversion of whole gene			*FOXO1B*	
**HsInv0340**	Gene disruption		*LINC00395*		
**HsInv0344**	Breakpoints overlap genes within IRs	*SNX6*	*RP11-671J11.7*, antisense RNA *RP11-671J11.4*		small nuclear RNAs *RNU1-27P* and *RNU1-28P*

Inversion	Effect	Protein-coding genes	Long non-coding RNAs	Pseudogenes	Other
**HsInv0347**	Inversion of whole gene				Small nucleolar RNA *U3*
**HsInv0374**	Inversion of part of intron			*AC005562.1* (*SMURF2P1-LRRC37BP1* readthrough transcribed pseudogene)	
	Inversion of whole gene			*SH3GL1P2*	
	Breakpoints overlap genes within IRs			*RP11-271K11.6, LRRC37BP1*	
**HsInv0379**	Gene disruption	*ZNF257*		*RP11-420K14.1*	
	Inversion of whole gene	*ZNF100, ZNF43, ZNF208*	*RP11-420K14.8, AC003973.4*	*MTDHP2, MTDHP3, MTDHP4, VN1R84P, RP11-420K14.6, BRI3BPP1, BNIP3P27, BNIP3P28*	miRNAs *AC092364.2* and *AC092364.4*
**HsInv0389**	Inversion of whole gene	*FLNA, EMD*			
**HsInv0393**	Breakpoints overlap genes within IRs	*ARMCX6*		*ARMCX7P*	
**HsInv0396**	Breakpoints overlap genes within IRs	*PABPC1L2A, PABPC1L2B*	antisense RNAs *PABPC1L2B-AS1* and *RP11-493K23.4*		
**HsInv0409**	Inversion of part of intron	*NLGN4X*			
**HsInv1051**	Gene disruption			*CCDC144B*	
	Breakpoints overlap genes within IRs	*PRPSAP2*		*AC107982.4*	small non-coding RNAs *RN7SL639P* and *RN7SL627P*
	Inversion of whole gene	*TBC1D28, ZNF286B, TRIM16L, FBXW10, TVP23B*	*CTD-2145A24.3*	*RP11-815I9.3, AC026271.5, FOXO3B , UBE2SP2, RP11-815I9.5, TRIM16L*	short non-coding RNA *RP11-815I9.4*
**HsInv1124**	Breakpoints overlap genes within IRs		*FAM225A, FAM225B*		

Supplementary Table 4. Potential phenotypic effects of inversions from GWAS data. Inversion effects were estimated through linkage disequilibrium (LD) with GWAS signals reported in the GWAS Catalog (http://www.ebi.ac.uk/gwas/) or GWASdb (http://jjwanglab.org/gwasdb) databases with a *P* value of less than 1×10^{-4}. Because each study is focused on individuals with different ancestry, we included in the analysis only inversions in high LD ($r^2 \geq 0.8$) with the GWAS variants in the studied population, the closest one available (e.g. TSI for Sardinian, JPT for Japanese, CHB for Han Chinese or Singapore Chinese, and GIH for South Asian, Indian or Bangladeshi) or the same population group (e.g. EUR for Ashkenazi, Framingham, British, Caucasian or Hutterite, and EAS for Korean). Finally, if studied populations were from different continents or not specified, we used the LD in the global population (GLB). References to each of the GWAS studies are indicated with the same number as in the Supplementary References list or with the dbGAP accession number.

Inversion	Database	GWAS variant	Chr	Position (hg19)	Population of study	Inv. LD (r^2) (Population)	GWAS *P* value	Phenotypic trait and reference
**HsInv0006**	GWAS Catalog	rs16937	chr1	205035455	Ashkenazi Jewish	0.811 (EUR)	5.00×10^{-7}	Schizophrenia[15]
**HsInv0058**	GWAS Catalog	rs2844665	chr6	31006855	European	1 (EUR)	3.00×10^{-7}	Drug-induced Stevens-Johnson syndrome or toxic epidermal necrolysis (SJS/TEN)[16]
**HsInv0004**	GWASdb	rs2488411	chr1	197658799	British	0.872 (EUR)	3.83×10^{-4}	Height[17]
**HsInv0004**	GWASdb	rs1775456	chr1	197733055	European	1 (EUR)	3.00×10^{-7}	Asthma[18]
**HsInv0004**	GWASdb	rs1924518	chr1	197738327	European	1 (EUR)	2.90×10^{-4}	Body mass index (asthmatics)[19]
**HsInv0006**	GWASdb	rs12142514	chr1	205122529	European	1 (EUR)	2.68×10^{-5}	Glaucoma (primary open-angle)[20]
**HsInv0006**	GWASdb	rs10900468	chr1	205163057	Not specified	0.881 (GLB)	5.30×10^{-5}	Blood pressure (dbGAP pha003046)
**HsInv0006**	GWASdb	rs10900468	chr1	205163057	Not specified	0.881 (GLB)	9.14×10^{-5}	Blood pressure (dbGAP pha003048)
**HsInv0031**	GWASdb	rs2937145	chr16	85190230	European	0.981 (EUR)	2.02×10^{-6}	Alzheimer's disease[21]
**HsInv0045**	GWASdb	rs465446	chr21	28022267	Caucasian	0.971 (EUR)	5.79×10^{-4}	Response to TNF antagonist treatment[22]
**HsInv0045**	GWASdb	rs366384	chr21	28024225	European	0.986 (EUR)	6.50×10^{-6}	Urinary metabolites[23]
**HsInv0058**	GWASdb	rs2844665	chr6	31006855	European	1 (EUR)	3.00×10^{-7}	Stevens-Johnson syndrome and toxic epidermal necrolysis (SJS-TEN)[16]
**HsInv0058**	GWASdb	rs2517538	chr6	31013541	Korean	1 (EAS)	2.60×10^{-5}	Height[24]
**HsInv0058**	GWASdb	rs2517538	chr6	31013541	Hutterite	0.964 (EUR)	2.07×10^{-21}	Lymphocyte counts[25]
**HsInv0063**	GWASdb	rs10269258	chr7	70440091	European	1 (EUR)	1.60×10^{-5}	Urinary metabolites[23]
**HsInv0098**	GWASdb	rs10510717	chr3	41332490	Framingham	0.882 (EUR)	5.00×10^{-5}	Volumetric brain MRI[26]
**HsInv0098**	GWASdb	rs1487569	chr3	41368428	Not specified	0.804 (GLB)	9.44×10^{-5}	Coronary artery disease[27]
**HsInv0098**	GWASdb	rs9311269	chr3	41374621	European	0.922 (EUR)	2.62×10^{-5}	Statin-induced myopathy[28]
**HsInv0409**	GWASdb	rs5916341	chrX	6135980	Not specified	1 (GLB)	2.47×10^{-4}	Amyotrophic lateral sclerosis[29]

Supplementary Methods

Inversion genotyping by MLPA and iMLPA

The multiplex ligation-dependent probe amplification (MLPA) technique enables the specific detection of a region of interest by using a pair of oligonucleotide probes (left and right probes) that hybridize contiguously to the target genome sequence in order to be ligated together in a subsequent step. The probes include a variable stuffer sequence and the sequence of the forward or reverse common primers, which are used for the simultaneous amplification of fragments of different sizes formed by the ligation of the left and right probes, and their detection by capillary electrophoresis[30]. In order to genotype at the same time multiple inversions with IRs or other repetitive sequences at the breakpoints, we developed a new method based on inverse PCR and MLPA that we termed inverse MLPA (iMLPA). iMLPA differs from normal MLPA by the addition of several extra initial steps that are necessary to obtain an orientation-specific unique target sequence for these inversions before probe hybridization.

The iMLPA protocol optimization was carried out by comparison with the known genotypes from the panel of nine individuals in which the inversions had been previously validated[2] (Supplementary Data 1). A detailed description of iMLPA steps can be found in the patent application EP13382296.5[31]. Briefly, 400 ng of genomic DNA of each sample were first digested overnight at 37ºC in six separated 20-µl reactions with 5 U of the appropriate restriction enzyme (*EcoRI, HindIII, SacI* or *BamHI* from Roche, and *NsiI* or *BglII* from New England Biolabs), followed by restriction enzyme inactivation for 15 min at 65ºC (with the exception of *BglII* that was inactivated for 20 min at 80ºC). Next, DNA self-ligation was performed overnight for 16-18 hours at 16°C by mixing together the six restriction enzyme digestions with 1x ligase buffer and 400 U of T4 DNA Ligase (New England Biolabs) in a total volume of 640 µl (resulting in an optimal concentration of 0.625 ng/µl of the DNA fragments generated by each restriction enzyme). Then, the ligase was inactivated and the DNA was fragmented by heating at 95ºC for 5 min, purified with the ZR-96 DNA Clean & Concentrator™-5 kit (Zymo Research) and resuspended in 7.5 µl of water.

The last step was the regular MLPA assay using the SALSA MLPA kit (MRC-Holland), according to the manufacturer instructions with minor modifications. In particular, the ligated DNA was denatured at 98ºC for 1.5 min, and probe hybridization was carried out adding 1.5 µl of our iMLPA probe mix (Supplementary Data 10) plus 1.5 µl of SALSA MLPA Buffer (MRC-Holland) and incubating for 1.5 min at 95ºC and 16 hours at 60ºC. Ligation of adjacent probes was then performed for 25 min at 54ºC by adding 25 µl water, 1 µl SALSA Ligase 65, 3 µl Ligase Buffer A and 3 µl Ligase Buffer B (MRC-Holland). After ligase inactivation (5 min at 95ºC), PCR amplification of ligated probes was performed separately in three groups of 8-9 inversions (Supplementary Data 10) using a common reverse primer and one of three forward primers labeled with a different fluorochrome (FAM, VIC or NED) (Supplementary Data 11). Each PCR was done in 25 µl with 5-6 µl of the iMLPA hybridization-ligation reaction, 2 µl SALSA PCR buffer (MRC-Holland), 0.25 mM each dNTP, 0.2 µM each primer, 1 µl PCR buffer without $MgCl_2$ (Roche), and 2.5 U of Taq DNA polymerase (Roche). PCR conditions were 95°C for 15 sec, 47 cycles of 95°C for 30 sec, 60°C for 30 sec and 72°C for 60 sec, and final extension at 72°C for 25 min. Finally, 0.67 µl of the three PCRs of each sample were mixed together, analyzed by capillary electrophoresis using an ABI PRISM 3130 Genetic Analyzer (Applied Biosystems), and the peaks were visually inspected using the GeneScan version 3.7 software (Applied Biosystems). For the regular MLPA, the process was identical with the exception that it started directly at the denaturation step of 100-150 ng of genomic DNA in 5 µl for 5 min at 98°C and that the ligated probes

were amplified in only two multiplex PCRs with 8-9 inversions each (Supplementary Data 10). In both cases all the successive reactions were carried out in a 96-well plate format to maximize speed and throughput and, with the exception of those used for optimization of the technique, only one MLPA or iMLPA reaction was done for every sample.

Visualization of inversion haplotypes and quantification of recurrence events

Reticulated networks are able to accommodate past recombination events, but each sequence is reduced to a node or edge, making it difficult to understand at the same time haplotype relationships and the spatial distribution of nucleotide changes along the sequence. Therefore, apart from building Median-Joining networks[32], we devised our own way to represent the similarities between haplotypes, named integrated haplotype plot (iHPlot), which are similar to the Haplostrips plots that have been recently developed independently[33]. Specifically, distances between simplified haplotypes after removing singleton positions were computed as the number of pairwise differences and were clustered with the UPGMA average method implemented in R[34] base function hclust. The corresponding dendrogram was then created using ggdendro R package[35] and all the information was integrated with a custom R script using ggplot2 and cowplot packages[36]. iHPlots were applied to the phased 1000GP Ph1 haplotypes of the inverted region and the imputed 1000GP Ph3 haplotypes based on inversion tag SNPs or on homozygotes for each orientation. For 1000GP Ph3 data, we used only accessible SNPs (excluding indels) according to the pilot accessibility mask that includes more SNPs than the strict mask[37]. In addition, besides the inverted region, whenever possible, we extended the analysis to the non-recombining region flanking the breakpoints (excluding associated indels and IRs) to increase the resolution of haplotype discrimination.

To determine more reliably the evolutionary history of each inversion, we combined the information from the different strategies for phasing and visualization of the inverted region haplotypes: 1) Median-joining networks of 1000GP Ph1 phased data; 2) iHPlots of 1000GP Ph1 phased data; and 3) iHPlots of 1000GP Ph3 published haplotypes (including the flanking non-recombining region if available). Moreover, HapMap phased data was used to confirm 1000GP results, although in many cases there was information from just a few SNPs. All inversions could be analyzed by at least some of the method combinations, except HsInv0041, which did not have enough variants and was excluded. Results of inversions with perfect tag variants ($r^2 = 1$) were determined mainly from the extended 1000GP Ph3 haplotypes, but consistent conclusions were obtained in the different analyses. The only exceptions were a few phasing errors by PHASE 2.1[3] in 1000GP Ph1 data in several inversions and a likely imputation error in HsInv0409 *O2*/*O2* individual NA20530 in 1000GP Ph3 (in which one of the haplotypes is typical of *O1* chromosomes, whereas in 1000GP Ph1 both haplotypes belong clearly to the *O2* group).

On the other hand, the estimation of recurrence events for inversions without tag variants relied mainly in the analysis of the iHPlots from phased 1000GP Ph1 haplotypes, since they contain all genotyped individuals in common, although there could be more phasing errors in inversion heterozygotes. First, we defined the putative original inversion event based on the ancestral allele information, the haplotype diversity within each orientation, and the frequency and geographical distribution of haplotypes, tending to favor as the first event those occurring in Africa. Next, we conservatively identified additional inversion or re-inversion events in differentiated clusters of haplotypes with both orientations. In order to consider that there has been inversion recurrence, these clusters have to differ from all other ones, and especially from those with the same orientation as the

potential recurrence event, by three or more sequence changes along most of the inverted region (and spanning at least 2 kb). Therefore, the presence of these nucleotide differences cannot be explained easily by other mechanisms, such as gene conversion or sequence errors. Direction of recurrence events was defined based on the relationship between the clusters and the frequency of the haplotypes with each orientation (Supplementary Data 3). Possible phasing errors in inversion heterozygotes were checked manually by determining if switching the orientation of both haplotypes still supports unequivocally the existence of recurrence. The same analysis was also repeated with 1000GP Ph3 iHPlots, in which just the orientation from haplotypes of *O1* and *O2* homozygotes is assigned, and only those clear recurrence events not invalidated with the new data were considered. It is important to take into account that since recurrence detection relies on differentiated haplotype clusters, it is not possible to distinguish more than one event within a cluster and there is a bias to predict more potential recurrence in larger inversions with more variants. For example, in six of the smallest NAHR inversions, *O1* and *O2* haplotypes are too similar to identify individual recurrence events (Supplementary Data 3). In two others (HsInv0124, HsInv0397), most *O1* and *O2* haplotypes belong to the same big cluster with just few differences between them and no clear recurrence can be identified. As a consequence, these results have to be interpreted with caution.

In the case of HsInv0832, we gathered publicly available information of the chr. Y haplogroups of 232 of the 282 genotyped males from different sources[38–43], as listed in Supplementary Data 4. Most of these studies determined also the evolutionary relationship between the chr. Y haplogroups, which were largely consistent and are shown in Fig. 3 in a simplified genealogical tree using the branch lengths of Poznik *et al.*[43]. This allowed us to identify with confidence five independent inversion events in the HsInv0832 region, assuming the most parsimonious scenario. HsInv0832 inversion rate was estimated dividing the number of inversion events (*n*) by the number of generations (*g*) encompassed in the phylogeny that relates the 217 Y-chromosomes for which sequence data was available[43]. To estimate *g*, we used the data from the B-T branch split to the leaves from Poznik *et al.*[43], including a B-T branch split time of 105.8 kya, a total number of mutations in all branches involved in the phylogeny that relates those 217 males of 17,332, and an average number of mutations of all branches of 784.57, plus a generation time of 25 years as in Repping *et al.*[44]. This results in 93,489 generations and an inversion rate of 5.35×10^{-5} per generation. In addition, to have another estimate of the inversion rate, we also used the approach of Hallast *et al.*[45], which was based on Repping *et al.*[44] that resequenced 80 kb in 47 Y chromosomes covering most major branches of the phylogenetic tree to obtain the nucleotide divergence in an unbiased manner. According to their data, we estimated a lower and upper bound of *g* of 127,467-336,533 generations by calculating the maximum (631) and minimum (239) total number of mutations spanning all the informative branches and an average number of mutations to the root in the different branches of 8.85, assuming a divergence time of 118 kya and a generation time of 25 years[44,45]. This yields an inversion rate of $1.48\text{-}3.92 \times 10^{-5}$, which is quite similar to the previous one.

Bioinformatic analysis of inversion orientation in non-human primate genomes

The bioinformatic analysis of inversion orientation in the available genome assemblies of chimpanzee (panTro5), gorilla (gorGor5), orangutan (ponAbe2) and rhesus macaque (macRhe8) was done using an automated bash script based on the command-line blat tool (v35x1)[46]. For each inversion, three separate hg18 sequences were extracted using twoBitToFa UCSC utility: the inverted region (or alternatively two separate internal 10-kb sequences adjacent to each breakpoint when the inverted region is longer than 20 kb) and the two 10-kb segments flanking each breakpoint outside the

inversion. We excluded the breakpoint intervals and their associated IRs and indels to avoid ambiguous mappings. Then, each sequence was aligned with blat to the genomes of interest, which were downloaded from the UCSC Genome Browser website in 2bit format. The longest hit was kept as the likely homologous region in the target assembly and orientation was defined as *O1* if all best hits mapped in the same strand, and as *O2* if the internal best hit(s) mapped in the opposite strand than those from the external sequences. As quality control, all best hits needed to be in the same scaffold or chromosome and the total span in the target assembly had to be 0.5-2-times that in hg18. In addition, in those cases in which the orientation could not be reliably defined or was inconsistent across species or with published data[47,48], results from the automated analysis were revised by aligning the sequences spanning the entire region from each assembly with the Gepard dotplot application[49] and Blast2seq[50], using default parameters.

Inversion age estimate

Inversion age was estimated from the net number of differences accumulated between sequences in opposite orientations. This number was obtained by subtracting from the mean pairwise nucleotide differences between *O1* and *O2* chromosomes, the expected average pairwise differences in the original population (before the generation of the inversion), which was approximated by the largest value of the average pairwise differences within sequences with the same orientation (either *O1* or *O2*). To ensure the maximum reliability of the divergence estimates, we considered all SNPs available in the extended 1000GP Ph3 haplotypes and sequence orientation was determined by the presence of tag variants or by using only *O1* and *O2* homozygous individuals. For two low-frequency inversions, HsInv0061 and HsInv1051, divergence could not be estimated because there were no tag variants in the analyzed region and all inversion carriers are heterozygous. A first age estimate was obtained by using a constant substitution rate of 10^{-9} changes per base-pair per year[51]. Moreover, in order to control for local differences in substitution rates, we obtained two additional local estimates from the divergence between human and chimpanzee or gorilla genomes, considering, respectively, a split time of 6 and 8 million years in each case (Supplementary Data 6). Pairwise LASTZ alignments[52] of human hg19 assembly with chimpanzee (assembly CSAC 2.1.4/panTro4) and gorilla (assembly gorGor3.1) genomes were retrieved from ENSEMBL GRCh37 portal[53], using the Compara Perl API[54]. We then used Kimura's two-parameter substitution model[55] to calculate the divergence between human and outgroup assemblies in the same inversion region analyzed above, after removing alignment gaps and non-syntenic alignment blocks. Alignments shorter than 1 kb were discarded, including the missing chimpanzee alignment for inversion HsInv0045 due to a deletion of the whole region and both outgroup alignments for inversion HsInv0041.

Simulation of inversion detection ascertainment bias

Given the heterogeneous origin of the inversions included in the study (Supplementary Data 1), to take into account the effect of ascertainment bias associated to inversion detection, we simulated two different processes using a bash and R[34] pipeline: one for the 38 autosomal or chr. X inversions detected from the fosmid paired-end mapping (PEM) data of nine individuals[56], and another for the six inversions detected exclusively by comparison of two genome assemblies[57]. First, we built panels from 1000GP individuals with matching demographic and gender composition to the detection samples. For the PEM study, corresponding to eight females and one male with African (4 YRI), East Asian (1 CHB and 1 JPT) and European ancestry (2 CEU and presumably European individual NA15510)[56,58], we were able to use all the original individuals except for NA19240 and NA15510, which were replaced with NA18502 and NA12717. For the genome comparison study, we used a

randomly selected European male (NA12872) and selected all SNPs that contained the alternative allele. Variants were filtered from 1000GP vcf files (ftp://ftp.1000genomes.ebi.ac.uk/vol1/ftp/release/20130502) with bcftools v1.7 view command[59]. Additional filters were applied to the SNPs to simplify comparisons (keeping only SNPs with assigned ID and ancestral allele in 1000GP vcf files), to use only putatively neutral variants (conservation GERP score[60] below 2 in functionally annotated 1000GP vcf files ftp://ftp-trace.ncbi.nih.gov/1000genomes/ftp/release/20130502/supporting/functional_annotation/unfiltered/), and to ensure high SNP quality (accessible according to the 1000GP Ph3 strict accessibility mask). However, the effect of these extra filters on the final frequency distribution was negligible, affecting just <1.5% of the detectable SNPs, which have similar average frequency to the rest of SNPs.

Second, we simulated the detection process of the inversions with the methods employed. This step was only simulated for the PEM data, since the limitations of inversion detection by assembly comparison are likely independent of variant frequency (and instead probably just related to repeat content and complexity of the genomic region). PEM detection, on the other hand, is affected by the sizes of the inversion and the IRs at the breakpoints, both of which limit the number of PEMs supporting it. To that end, we modeled the detection of an inversion that is present in the PEM panel as a function of these two characteristics and the number of chromosomes with the alternative orientation. Specifically, the probability of having two discordant PEMs in the whole panel (the minimum number necessary to detect an inversion) was calculated by a Poisson distribution with a lambda parameter equal to the expected number of discordant PEMs (E(disc)). Following Equation 1 in Lucas-Lledó *et al.*[61], E(disc) for the two breakpoints of a given inversion (inv) and IR (ir) size, considering the average PEM insert length (ins) and read length (read), was estimated as:

$$E(\text{disc}) = 2\,\frac{min(\text{inv} - \text{read}, \text{ins} - 2\ \text{read} - \text{ir})}{g}\,n\,f$$

where g is the sequenced haploid genome size (approximated to 3 Gb), n is the total number of fosmids sequenced[56,62], and f is the fraction of chromosomes carrying the mutation in the nine individuals analyzed. For each PEM inversion, a custom R script was used to generate a matching random sample of 10,000 SNPs. These SNPs were selected according to the detection probability of the SNPs based on their frequency in the PEM panel and the inversion characteristics, including the chromosome type (autosomes or chr. X).

Frequency differences between populations (F_{ST})

To calculate F_{ST}[63], we created vcf files containing the inversion genotypes for the 434 individuals common to 1000GP Ph3 and used the --weir-fst-pop option from vcftools (v0.1.15). F_{ST} values were obtained for each pair of populations within the same population group, each pair of population groups, and globally. Genome-wide F_{ST} null distributions were obtained from 1000GP Ph3 bialellic SNPs polymorphic in the same 434 individuals that are accessible according to the strict criteria and have a defined ancestral allele. To control F_{ST} dependence on chromosome type and allele frequency, empirical P values for each inversion and comparison were estimated as the fraction of the SNP distribution (including always a minimum of 10,000 SNPs) from the same chromosome type (autosome or chr. X) and global MAF bin (from 0 to 0.5 in 0.05 increases) as the inversion with equal or larger F_{ST} (Supplementary Data 7). Reduced levels of population differentiation are sometimes interpreted as evidence of balancing selection. However, power to detect the extreme low F_{ST} values

was very low. Global population differentiation for all inversions together was measured by a hierarchic analysis of molecular variance (AMOVA) according to geographic criteria using Arlequin v3.5[64]. Resulting variation was mainly due to the difference between the three continental (CT) groups for both autosomal inversions (F_{ST} = 0.13, P < 0.0001; F_{CT} = 0.11, P < 0.0001; F_{SC} = 0.03, P = 0.02) and chr. X inversions (F_{ST} = 0.24, P < 0.0001; F_{CT} = 0.20, P < 0.0001; F_{SC} = 0.05, P < 0.0001).

Linked site frequency spectrum (LSFS) selection tests

For LSFS tests we used a simplified version of the tests, i.e. weights were chosen computing the covariance in the approximation of unlinked sites, and we assumed strong selection coefficients in two scenarios: (1) classical selective sweep (positive selection); and (2) long-term balancing selection. The frequency spectrum of variants closely linked to the inversion, including their linkage pattern (nested or disjoint) with the inverted allele[65], was calculated in relatively-small non-overlapping windows of 3 kb in order to reduce the effects of recombination within each window on the empirical null spectrum. The windows tested were localized either within the inversion or the non-recombining flanking regions and skipped the breakpoint interval and IRs to avoid errors from associated indels or incorrect short-read mappings. The autosome-wide empirical spectrum was computed on windows of the same size (3 kb) around all autosomal SNPs. The LSFS was calculated from biallelic 1000GP Ph3 SNPs in the 434 samples with inversion genotypes. We removed from the analysis all SNPs with a GERP score[60] higher than 2 to reduce the effect of linked selection, as well as those SNPs within 0.5 Mb of any of the inversions in our dataset, since their dynamics could be heavily influenced by the inversion itself. Tests were conditioned on the inversion frequency in the different populations. For each test distribution conditioned on minor allele counts of at least 6, a local cubic smoothing was finally applied to the frequency dependence of the distribution, considering derived allele counts between +5 and -5 with respect to that of the inversion. In addition, to control for the complex demographic history of human populations, we used the empirical autosome-wide first and second moments of the empirical linked frequency spectrum of SNPs in each population as a substitute for the null spectrum.

Edgington's method[66] was used to combine the P values of the same windows of each population. Combining the results across different windows of an inversion is complicated by the correlation of their P values, since in the absence of recombination they share the same evolutionary history. We dealt with this in two ways. The first approach (conservative) was to assume an arbitrary dependence between windows, and compute the False Discovery Rate (FDR) correcting for multiple correlated testing via Benjamini-Hochberg-Yakutieli[67] for each inversion separately and for all inversions together (in the latter case, HsInv0379 was removed from the analysis due to its size and unbalanced contribution to the statistical noise) (Supplementary Data 7). The second approach (approximate) is to approximate the joint distribution across correlated windows as a multidimensional Gaussian distribution by: (1) applying a Gaussian transformation to the P values; (2) computing the empirical correlation across all pairs of windows of the same inversion; (3) computing the average Gaussian score for each inversion; (4) building an equicorrelated matrix of the same size as the number of windows in the inversion, with elements equal to 1 on the diagonal and to the empirical correlation off the diagonal; and (5) comparing the average Gaussian score with the average score extracted from a multidimensional Gaussian distribution with covariances distributed as the equicorrelated matrix. This approach was applied both to each population separately and to the combined P values from all populations (Supplementary Data 7).

Non-central deviation (NCD) selection tests

NCD statistics were adapted to test long-term balancing selection acting on autosomal and chr. X inversion regions. NCD1 detects site frequency spectrum shifts towards an equilibrium frequency as expected under balancing selection, whereas NCD2 incorporates also information on polymorphism density and is most powerful to detect long-term balancing selection[68]. NCD1 and NCD2 were computed genome-wide as previously described[68] using overlapping windows of 2 kb (with 1 kb step), which fit well the size of the smaller inversions, and three target frequencies (0.3, 0.4 and 0.5). Human polymorphism data was obtained from 1000GP Ph3 SNPs from all individuals of the seven studied populations accessible according to the pilot accessibility mask, and human-chimpanzee differences were obtained from the hg19-panTro4 alignments available at the UCSC Genome Browser[46]. Windows of the 44 inversions were defined with the same criteria as in the LSFS test, including the inverted and flanking non-recombining region, while avoiding breakpoint, IR and indel intervals. Nine inversions did not have any window passing the filtering criteria and were not analyzed (HsInv0031, HsInv0041, HsInv0045, HsInv0055, HsInv0061, HsInv0072, HsInv0344, HsInv0409, and HsInv1124).

A raw empirical *P* value was assigned to each inversion window corresponding to their quantile in the null genome-wide distribution of the statistic in that population computed with the target frequency most similar to the inversion global MAF[68], and the lowest *P* value of all the windows for each inversion and population was selected. To correct for the fact that some inversions have more than one window, we then sampled 1,000 sets of regions of equal size and from the same chromosome as each of the inversions, selected the lowest *P* value of all the windows of each region, and obtained the empirical distribution of minimum *P* values equivalent to that of the inversion. Finally, size-corrected *P* values for each inversion and population were estimated from the quantile in the corresponding minimum-*P*-value distribution (Supplementary Data 7). Since balancing selection signals are expected to be shared across multiple populations[68], we chose as candidates those inversions with three or more populations with size-corrected *P* values < 0.01 (strong candidates) or *P* values < 0.05 (weak candidates). The main limitation of these tests is that, by reducing recombination, inversions may affect the expected empirical distribution. For example, inversions increase variance in the SFS or the age of alleles. Nevertheless, the reduced recombination means stronger effects of background selection, which results in lower levels of diversity and younger alleles, which are the opposite to the signatures detected by the NCD statistics. An additional limitation is that the signatures of balancing selection could be due to any SNP within the windows, rather than the inversion itself. However, the functional effects of the inversion are expected to be much stronger than those of a single nucleotide change.

Validation of lymphoblastoid cell lines (LCLs) gene-expression analysis results

We employed different strategies to confirm the reliability of the results of the gene expression analysis from LCLs, which are summarized in Supplementary Fig. 4C-F. In particular, we compared our results with those of two additional commonly-used eQTL mapping methods: the one described by the GTEx Project[6] and edgeR-limma[7,8]. In the GTEx analysis, RPKM values were quantile normalized across all samples and gene/transcript expression levels were subsequently adjusted by rank-based inverse normal transformation per each gene and transcript. In this case, technical confounding variation was accounted with the PEER software[69]. The number of technical covariates was chosen to optimize eQTL identification by maximizing consistent eQTL calls and minimizing differences between GTEx and QTLtools pipelines, but avoiding overfitting the model. We tested up to

the top 60 expression-derived PEER factors and 60 principal components of the PCA, taken in groups of 5 in decreasing order of the variance explained, and determined the optimal number according to the results overlap. Linear regressions were then done with FastQTL v2.0[70], including the selected PEER factors (for gene and transcript analysis, respectively, 5 and 20 in the experimental dataset and 35 and 55 in the imputed dataset), gender, and the three population principal components as covariates. In the edgeR-limma workflow, raw read counts were corrected by library size in counts per million. Genes and transcripts that passed the expression-level cutoff (0.1 counts per million in at least two samples) were normalized with trimmed mean of M-values (TMM)[71] and transformed with voom[7]. Next, limma fit an additive linear model to contrast differentially expressed genes across genotypes, including gender, population and sequencing laboratory as covariates. Other potential batch effects were uncovered with the SVA package (1 and 2 for experimental and imputed sets, respectively)[72]. All *P* values were corrected by Storey & Tibshirani FDR[73].

As an independent replication of these results, we also examined the available gene-expression data from blood samples of ~2,000 Estonian individuals obtained by hybridization with Illumina HumanHT-12 v3.0 Gene Expression BeadChip arrays. In this case, we checked directly the effects of 1,541 SNPs that were in high LD ($r^2 \geq 0.8$) with 33 inversions either globally (27) or just in Europeans (6). These SNPs were already imputed in Estonian samples based on 1000GP Ph1 variants. In total, six potential inversion-eQTL effects were identified in this study in blood (FDR < 5%): HsInv0006 and *DSTYK*; HsInv0058 and *HLA-E* and *HLA-C*; HsInv0095 and *SPP1*; HsInv0201 and *FBXO38*; and HsInv0209 and *FOLR3*. Of those, five were also found in the GTEx or GEUVADIS data, which represents a good degree of consistency considering the different expression quantification platforms and analysis methods used.

Integrative analysis of functional and selection evidence

Overlap of functional and selection signals for the 44 autosomal and chr. X inversions analyzed was calculated by a Fisher's exact test of independence. To reduce possible spurious signals, we focused on selection signatures calculated on the inversion itself (excluding NCD1 and NCD2 test results) and all functional effects except those from GWAS data, which in most cases are related to diseases and could have detrimental consequences during evolution. Criteria for classification of strong and weak selection and functional evidence are explained in Supplementary Data 7 or Fig. 2. The association was replicated considering only the strongest functional effects and selection signals for the 44 inversions (Fisher's exact test $P = 0.0130$) or just the 21 inversions with perfect tag SNPs that were included in most analyses, which comprise all NH inversions, except HsInv0102, plus HsInv0040 (Fisher's exact test $P = 0.0300$).

Supplementary References

1. Sudmant, P. H. *et al.* An integrated map of structural variation in 2,504 human genomes. *Nature* **526,** 75–81 (2015).
2. Martínez-Fundichely, A. *et al.* InvFEST, a database integrating information of polymorphic inversions in the human genome. *Nucleic Acids Res.* **42,** D1027-32 (2014).
3. Stephens, M. & Donnelly, P. A comparison of bayesian methods for haplotype reconstruction from population genotype data. *Am. J. Hum. Genet.* **73,** 1162–9 (2003).
4. Howie, B. N., Donnelly, P. & Marchini, J. A Flexible and Accurate Genotype Imputation Method for the Next Generation of Genome-Wide Association Studies. *PLoS Genet.* **5,** e1000529 (2009).
5. Delaneau, O. *et al.* A complete tool set for molecular QTL discovery and analysis. *Nat. Commun.* **8,** 15452 (2017).
6. GTEx Consortium. Genetic effects on gene expression across human tissues. *Nature* **550,** 204–13 (2017).
7. Ritchie, M. E. *et al.* limma powers differential expression analyses for RNA-sequencing and microarray studies. *Nucleic Acids Res.* **43,** e47 (2015).
8. Robinson, M. D., McCarthy, D. J. & Smyth, G. K. edgeR: a Bioconductor package for differential expression analysis of digital gene expression data. *Bioinformatics* **26,** 139–40 (2010).
9. Hulsen, T., de Vlieg, J. & Alkema, W. BioVenn – a web application for the comparison and visualization of biological lists using area-proportional Venn diagrams. *BMC Genomics* **9,** 488 (2008).
10. Dobin, A. *et al.* STAR: ultrafast universal RNA-seq aligner. *Bioinformatics* **29,** 15–21 (2013).
11. Roberts, A., Pimentel, H., Trapnell, C. & Pachter, L. Identification of novel transcripts in annotated genomes using RNA-Seq. *Bioinformatics* **27,** 2325–9 (2011).
12. Robinson, J. T. *et al.* Integrative genomics viewer. *Nat. Biotechnol.* **29,** 24–6 (2011).
13. Machiela, M. J. & Chanock, S. J. LDlink: a web-based application for exploring population-specific haplotype structure and linking correlated alleles of possible functional variants. *Bioinformatics* **31,** 3555–7 (2015).
14. Purcell, S. *et al.* PLINK: a tool set for whole-genome association and population-based linkage analyses. *Am. J. Hum. Genet.* **81,** 559–75 (2007).
15. Goes, F. S. *et al.* Genome-wide association study of schizophrenia in Ashkenazi Jews. *Am. J. Med. Genet. Part B Neuropsychiatr. Genet.* **168,** 649–59 (2015).
16. Génin, E. *et al.* Genome-wide association study of Stevens-Johnson Syndrome and Toxic Epidermal Necrolysis in Europe. *Orphanet J. Rare Dis.* **6,** 52 (2011).
17. Strachan, D. P. *et al.* Lifecourse influences on health among British adults: effects of region of residence in childhood and adulthood. *Int. J. Epidemiol.* **36,** 522–31 (2007).
18. Ferreira, M. A. R. *et al.* Identification of IL6R and chromosome 11q13.5 as risk loci for asthma. *Lancet* **378,** 1006–14 (2011).
19. Melén, E. *et al.* Genome-wide association study of body mass index in 23 000 individuals with and without asthma. *Clin. Exp. Allergy* **43,** 463–74 (2013).
20. Gibson, J. *et al.* Genome-wide association study of primary open angle glaucoma risk and quantitative traits. *Mol. Vis.* **18,** 1083–92 (2012).
21. Pérez-Palma, E. *et al.* Overrepresentation of glutamate signaling in Alzheimer’s disease: network-based pathway enrichment using meta-analysis of genome-wide association studies. *PLoS One* **9,** e95413 (2014).
22. Plant, D. *et al.* Genome-wide association study of genetic predictors of anti-tumor necrosis factor treatment efficacy in rheumatoid arthritis identifies associations with polymorphisms at seven loci. *Arthritis Rheum.* **63,** 645–53 (2011).
23. Suhre, K. *et al.* A genome-wide association study of metabolic traits in human urine. *Nat. Genet.* **43,** 565–9 (2011).
24. Cho, Y. S. *et al.* A large-scale genome-wide association study of Asian populations uncovers

genetic factors influencing eight quantitative traits. *Nat. Genet.* **41,** 527–34 (2009).

25. Cusanovich, D. A. *et al.* The combination of a genome-wide association study of lymphocyte count and analysis of gene expression data reveals novel asthma candidate genes. *Hum. Mol. Genet.* **21,** 2111–23 (2012).
26. Seshadri, S. *et al.* Genetic correlates of brain aging on MRI and cognitive test measures: a genome-wide association and linkage analysis in the Framingham Study. *BMC Med. Genet.* **8 Suppl 1,** S15 (2007).
27. Samani, N. J. *et al.* Genomewide Association Analysis of Coronary Artery Disease. *N. Engl. J. Med.* **357,** 443–53 (2007).
28. Isackson, P. J. *et al.* Association of common variants in the human eyes shut ortholog (EYS) with statin-induced myopathy: evidence for additional functions of EYS. *Muscle Nerve* **44,** 531–8 (2011).
29. Schymick, J. C. *et al.* Genome-wide genotyping in amyotrophic lateral sclerosis and neurologically normal controls: first stage analysis and public release of data. *Lancet. Neurol.* **6,** 322–8 (2007).
30. Schouten, J. P. *et al.* Relative quantification of 40 nucleic acid sequences by multiplex ligation-dependent probe amplification. *Nucleic Acids Res.* **30,** e57 (2002).
31. Cáceres, M., Villatoro, S. & Aguado, C. Inverse Multiplex Ligation-dependent Probe Amplification (iMLPA), an in vitro method of genotyping multiple inversions. (2015).
32. Bandelt, H. J., Forster, P. & Röhl, A. Median-joining networks for inferring intraspecific phylogenies. *Mol. Biol. Evol.* **16,** 37–48 (1999).
33. Marnetto, D. & Huerta-Sánchez, E. Haplostrips : revealing population structure through haplotype visualization. *Methods Ecol. Evol.* **8,** 1389–92 (2017).
34. R Core Team. R: A Language and Environment for Statistical Computing. (2017).
35. de Vries, A. & Ripley, B. D. ggdendro: Create Dendrograms and Tree Diagrams Using ‘ggplot2’. (2016).
36. Wickham, H. *ggplot2: Elegant Graphics for Data Analysis*. (Springer-Verlag New York, 2009).
37. The 1000 Genomes Project Consortium. A global reference for human genetic variation. *Nature* **526,** 68–74 (2015).
38. Wei, W. *et al.* A calibrated human Y-chromosomal phylogeny based on resequencing. *Genome Res.* **23,** 388–95 (2013).
39. Magoon, G. R. *et al.* Generation of high-resolution a priori Y-chromosome phylogenies using ‘next-generation’ sequencing data. *bioRxiv* 802 (2013). doi:10.1101/000802
40. Wang, C.-C. & Li, H. Discovery of Phylogenetic Relevant Y-chromosome Variants in 1000 Genomes Project Data. *arXiv* 1310.6590 (2013).
41. Van Geystelen, A., Decorte, R. & Larmuseau, M. H. D. Updating the Y-chromosomal phylogenetic tree for forensic applications based on whole genome SNPs. *Forensic Sci. Int. Genet.* **7,** 573–80 (2013).
42. Hallast, P. *et al.* The Y-Chromosome tree bursts into leaf: 13,000 high-confidence SNPs covering the majority of known clades. *Mol. Biol. Evol.* **32,** 661–73 (2015).
43. Poznik, G. D. *et al.* Punctuated bursts in human male demography inferred from 1,244 worldwide Y-chromosome sequences. *Nat. Genet.* **48,** 593–9 (2016).
44. Repping, S. *et al.* High mutation rates have driven extensive structural polymorphism among human Y chromosomes. *Nat. Genet.* **38,** 463–7 (2006).
45. Hallast, P., Balaresque, P., Bowden, G. R., Ballereau, S. & Jobling, M. A. Recombination dynamics of a human Y-chromosomal palindrome: Rapid GC-biased gene conversion, multi-kilobase conversion tracts, and rare inversions. *PLoS Genet.* **9,** e1003666 (2013).
46. Kent, W. J. *et al.* The Human Genome Browser at UCSC. *Genome Res.* **12,** 996–1006 (2002).
47. Aguado, C. *et al.* Validation and genotyping of multiple human polymorphic inversions mediated by inverted repeats reveals a high degree of recurrence. *PLoS Genet.* **10,** e1004208 (2014).
48. Vicente-Salvador, D. *et al.* Detailed analysis of inversions predicted between two human

genomes: errors, real polymorphisms, and their origin and population distribution. *Hum. Mol. Genet.* **26,** 567–81 (2017).

49. Krumsiek, J., Arnold, R. & Rattei, T. Gepard: a rapid and sensitive tool for creating dotplots on genome scale. *Bioinformatics* **23,** 1026–8 (2007).

50. Altschul, S. F., Gish, W., Miller, W., Myers, E. W. & Lipman, D. J. Basic local alignment search tool. *J. Mol. Biol.* **215,** 403–10 (1990).

51. Moorjani, P., Gao, Z. & Przeworski, M. Human germline mutation and the erratic evolutionary clock. *PLoS Biol.* **14,** e2000744 (2016).

52. Harris, R. S. Improved pairwise alignment of genomic DNA. (The Pennsylvania State University, 2007).

53. Yates, A. *et al.* Ensembl 2016. *Nucleic Acids Res.* **44,** D710-6 (2016).

54. Herrero, J. *et al.* Ensembl comparative genomics resources. *Database* **2016,** bav096 (2016).

55. Kimura, M. A simple method for estimating evolutionary rates of base substitutions through comparative studies of nucleotide sequences. *J. Mol. Evol.* **16,** 111–20 (1980).

56. Kidd, J. M. *et al.* Mapping and sequencing of structural variation from eight human genomes. *Nature* **453,** 56–64 (2008).

57. Levy, S. *et al.* The diploid genome sequence of an individual human. *PLoS Biol.* **5,** e254 (2007).

58. Korbel, J. O. *et al.* Paired-end mapping reveals extensive structural variation in the human genome. *Science* **318,** 420–6 (2007).

59. Li, H. A statistical framework for SNP calling, mutation discovery, association mapping and population genetical parameter estimation from sequencing data. *Bioinformatics* **27,** 2987–93 (2011).

60. Davydov, E. V *et al.* Identifying a high fraction of the human genome to be under selective constraint using GERP++. *PLoS Comput. Biol.* **6,** e1001025 (2010).

61. Lucas-Lledó, J. I. & Cáceres, M. On the power and the systematic biases of the detection of chromosomal inversions by paired-end genome sequencing. *PLoS One* **8,** e61292 (2013).

62. Tuzun, E. *et al.* Fine-scale structural variation of the human genome. *Nat. Genet.* **37,** 727–32 (2005).

63. Weir, B. S. & Cockerham, C. C. Estimating F-statistics for the analysis of population structure. *Evolution (N. Y).* **38,** 1358–70 (1984).

64. Excoffier, L. & Lischer, H. E. L. Arlequin suite ver 3.5: a new series of programs to perform population genetics analyses under Linux and Windows. *Mol. Ecol. Resour.* **10,** 564–7 (2010).

65. Ferretti, L. *et al.* The neutral frequency spectrum of linked sites. *Theor. Popul. Biol.* **123,** 70–9 (2018).

66. Edgington, E. S. An additive method for combining probability values from independent experiments. *J. Psychol.* **80,** 351–63 (1972).

67. Benjamini, Y. & Yekutieli, D. The control of the false discovery rate in multiple testing under dependency. *Ann. Stat.* **29,** 1165–88 (2001).

68. Bitarello, B. D. *et al.* Signatures of long-term balancing selection in human genomes. *Genome Biol. Evol.* **10,** 939–55 (2018).

69. Stegle, O., Parts, L., Piipari, M., Winn, J. & Durbin, R. Using probabilistic estimation of expression residuals (PEER) to obtain increased power and interpretability of gene expression analyses. *Nat. Protoc.* **7,** 500–7 (2012).

70. Ongen, H., Buil, A., Brown, A. A., Dermitzakis, E. T. & Delaneau, O. Fast and efficient QTL mapper for thousands of molecular phenotypes. *Bioinformatics* **32,** 1479–85 (2016).

71. Robinson, M. D. & Oshlack, A. A scaling normalization method for differential expression analysis of RNA-seq data. *Genome Biol.* **11,** R25 (2010).

72. Leek, J. *et al.* sva: Surrogate Variable Analysis. R package version 3.28.0 (2018).

73. Storey, J. D. & Tibshirani, R. Statistical significance for genomewide studies. *Proc. Natl. Acad. Sci.* **100,** 9440–5 (2003).

74. Hehir-Kwa, J. Y. *et al.* A high-quality human reference panel reveals the complexity and distribution of genomic structural variants. *Nat. Commun.* **7,** 12989 (2016).

75. Huddleston, J. *et al.* Discovery and genotyping of structural variation from long-read haploid genome sequence data. *Genome Res.* **27,** 677–85 (2017).

76. Sanders, A. D. *et al.* Characterizing polymorphic inversions in human genomes by single cell sequencing. *Genome Res.* **26,** 1575–87 (2016).

77. Li, L. *et al.* OMSV enables accurate and comprehensive identification of large structural variations from nanochannel-based single-molecule optical maps. *Genome Biol.* **18,** 230 (2017).

78. Audano, P. A. *et al.* Characterizing the major structural variant alleles of the human genome. *Cell* **176,** 663–75 (2019).

79. Chaisson, M. J. P. *et al.* Multi-platform discovery of haplotype-resolved structural variation in human genomes. *Nat. Commun.* **10,** 1784 (2019).

80. Martínez-Fundichely, A. *et al.* Accurate characterization of inversions in the human genome from paired-end mapping data with the GRIAL algorithm. *In prep.*

81. Lucas-Lledó, J. I., Vicente-Salvador, D., Aguado, C. & Cáceres, M. Population genetic analysis of bi-allelic structural variants from low-coverage sequence data with an expectation-maximization algorithm. *BMC Bioinformatics* **15,** 163 (2014).

82. Puig, M. *et al.* Functional impact and evolution of a novel human polymorphic inversion that disrupts a gene and creates a fusion transcript. *PLoS Genet.* **11,** e1005495 (2015).

83. Prüfer, K. *et al.* The complete genome sequence of a Neanderthal from the Altai Mountains. *Nature* **505,** 43–9 (2014).

84. Meyer, M. *et al.* A high-coverage genome sequence from an archaic Denisovan individual. *Science* **338,** 222–6 (2012).

85. Keller, A. *et al.* New insights into the Tyrolean Iceman's origin and phenotype as inferred by whole-genome sequencing. *Nat. Commun.* **3,** 698 (2012).

86. Olalde, I. *et al.* Derived immune and ancestral pigmentation alleles in a 7,000-year-old Mesolithic European. *Nature* **507,** 225–8 (2014).

87. Pantano, L., Armengol, L., Villatoro, S. & Estivill, X. ProSeeK: A web server for MLPA probe design. *BMC Genomics* **9,** 573 (2008).
