# Supplementary material for: Evolutionary and functional impact of common polymorphic inversions in the human genome
Source: Nat Commun. 2019 Sep 17;10:4222. doi: 10.1038/s41467-019-12173-x (PMC6748972; doi:10.1038/s41467-019-12173-x)
Supplement: Supplementary file 3 — Reporting Summary [file 41467_2019_12173_MOESM3_ESM.pdf]

nature research

Corresponding author(s): Mario Cáceres

Last updated by author(s): Aug 20, 2019

Reporting Summary

Nature Research wishes to improve the reproducibility of the work that we publish. This form provides structure for consistency and transparency in reporting. For further information on Nature Research policies, see Authors & Referees and the Editorial Policy Checklist.

Statistics

For all statistical analyses, confirm that the following items are present in the figure legend, table legend, main text, or Methods section.

n/a	Confirmed	
☐	☒	The exact sample size (*n*) for each experimental group/condition, given as a discrete number and unit of measurement
☐	☒	A statement on whether measurements were taken from distinct samples or whether the same sample was measured repeatedly
☐	☒	The statistical test(s) used AND whether they are one- or two-sided<br>*Only common tests should be described solely by name; describe more complex techniques in the Methods section.*
☐	☒	A description of all covariates tested
☐	☒	A description of any assumptions or corrections, such as tests of normality and adjustment for multiple comparisons
☐	☒	A full description of the statistical parameters including central tendency (e.g. means) or other basic estimates (e.g. regression coefficient) AND variation (e.g. standard deviation) or associated estimates of uncertainty (e.g. confidence intervals)
☐	☒	For null hypothesis testing, the test statistic (e.g. *F*, *t*, *r*) with confidence intervals, effect sizes, degrees of freedom and *P* value noted<br>*Give P values as exact values whenever suitable.*
☐	☒	For Bayesian analysis, information on the choice of priors and Markov chain Monte Carlo settings
☒	☐	For hierarchical and complex designs, identification of the appropriate level for tests and full reporting of outcomes
☐	☒	Estimates of effect sizes (e.g. Cohen's *d*, Pearson's *r*), indicating how they were calculated

Our web collection on statistics for biologists contains articles on many of the points above.

Software and code

Policy information about availability of computer code

Data collection	GeneMapper v3.7 (MLPA and iMLPA)
Data analysis	- R 3.4.0 or 3.3.0<br>- R packages: ggplot2 2.2.1, cowplot 0.9.2, ggdendro 0.1.20, robustbase 0.93.1, edgeR 3.14.0, limma 3.28.21, peer 1.0, preprocessCore 1.34.0, sva 3.20.0, qvalue 2.4.2, data.table 1.10.4, robustbase 0.92-8, cowplot 0.8.0, RColorBrewer 1.1-2, ggplot2 2.2.1<br>- bcftools 1.7 (htslib 1.7)<br>- plink v1.90<br>- PHASE 2.1<br>- NETWORK v4.6.1.3<br>- LDhat v2.2<br>- IMPUTE v2.3.2<br>- tabix & bgzip (htslib) 1.3.1<br>- STAR v2.4.2a<br>- RSEM v1.2.31<br>- FastQTL v2.0<br>- VCFtools v0.1.11<br>- QTLtools v1.1<br>- BioVenn<br>- Cufflinks v2.2.1<br>- Integrative Genomics Viewer v2.3.92<br>- Samtools v0.1.19<br>- bedtools v2.17.0

For manuscripts utilizing custom algorithms or software that are central to the research but not yet described in published literature, software must be made available to editors/reviewers. We strongly encourage code deposition in a community repository (e.g. GitHub). See the Nature Research guidelines for submitting code & software for further information.

Data

Policy information about availability of data

All manuscripts must include a data availability statement. This statement should provide the following information, where applicable:

- Accession codes, unique identifiers, or web links for publicly available datasets
- A list of figures that have associated raw data
- A description of any restrictions on data availability

All data described in this article are available in the Supplementary Information and in the InvFEST database (http://invfestdb.uab.cat/). In addition, inversion genotypes have been deposited in the dbVar database (https://www.ncbi.nlm.nih.gov/dbvar/) under accession number nstd169 [https://www.ncbi.nlm.nih.gov/dbvar/studies/nstd169/]. The source data underlying Figs. 2, 3a-c, 4a-c, 5a, c, e and 6a-b and Supplementary Figs. 1, 2a-b, 4a, f, 5 and 8a-c are provided as a Source Data file. All other relevant data is available upon request.

Field-specific reporting

Please select the one below that is the best fit for your research. If you are not sure, read the appropriate sections before making your selection.

☒ Life sciences ☐ Behavioural & social sciences ☐ Ecological, evolutionary & environmental sciences

For a reference copy of the document with all sections, see nature.com/documents/nr-reporting-summary-flat.pdf

Life sciences study design

All studies must disclose on these points even when the disclosure is negative.

Sample size	Samples were chosen to represent several human populations across different continents and they are commonly used in multiple human population and genomic analysis.
Data exclusions	No data were excluded from the analyses.
Replication	3377 MLPA/iMLPA inversion genotypes were compared with previous data, and 2160 inversion genotypes were validated by PCR/iPCR in this work.
Randomization	Samples were allocated in different analysis groups based on their inversion genotypes and those of other genetic variants.
Blinding	No blinding was performed since group allocation was done automatically based on sample genotypes that were determined independently.

Reporting for specific materials, systems and methods

We require information from authors about some types of materials, experimental systems and methods used in many studies. Here, indicate whether each material, system or method listed is relevant to your study. If you are not sure if a list item applies to your research, read the appropriate section before selecting a response.

Materials & experimental systems

n/a	Involved in the study	
☒	☐	Antibodies
☐	☒	Eukaryotic cell lines
☒	☐	Palaeontology
☒	☐	Animals and other organisms
☒	☐	Human research participants
☒	☐	Clinical data

Methods

n/a	Involved in the study	
☒	☐	ChIP-seq
☒	☐	Flow cytometry
☒	☐	MRI-based neuroimaging

Eukaryotic cell lines

Policy information about cell lines

Cell line source(s)	Coriell Cell Biorepositories (Camden, NJ, USA) (human cell lines) and private collections (non-human primate cell lines).
Authentication	Human cell lines were authenticated with the MSK microsatellite kit (Coriell Cell Repository, Camden, NJ, USA).
Mycoplasma contamination	Cell lines were not tested for mycoplasma contamination.
Commonly misidentified lines (See ICLAC register)	No commonly misidentified cell lines were used in this study.
