# Supplementary material for: Evolutionary and functional impact of common polymorphic inversions in the human genome
Source: Nat Commun. 2019 Sep 17;10:4222. doi: 10.1038/s41467-019-12173-x (PMC6748972; doi:10.1038/s41467-019-12173-x)
Supplement: Supplementary file 4 — Description of Additional Supplementary Files [file 41467_2019_12173_MOESM4_ESM.pdf]

Description of Additional Supplementary Files

File Name: Supplementary Data 1

Description: Main features of the 45 human polymorphic inversions analyzed in this study. Inversion breakpoint annotations using hg18 as the reference genome were revised and updated here from the information already available from the existing studies (listed in the Genotype validation column) and they are described in more detail in the InvFEST database. *O1* refers always to the orientation found in hg18 and previously labeled as standard (*Std*), whereas *O2* refers to the alternative orientation, previously labeled as inverted (*Inv*). ^ indicates that the inversion breakpoint is located between two adjacent positions. For those inversions flanked by inverted repeats (IRs) or with indels at the breakpoints, the main characteristics are shown, including the size, identity and type of the IRs and the position and size of indels (Del, deletion; Dup, Duplication; Ins, Insertion), using whenever possible the annotations for segmental duplications (SDs) and transposable elements (TEs) from the UCSC genome browser[46]. The information on inversion detection by different studies includes those of Levy et al.[57] and Kidd et al.[56] from which the inversions were originally selected, plus other studies in which at least two different individuals were analyzed. When necessary, the UCSC liftOver tool[46] was used to convert hg18 coordinates into hg19 or hg38. References to previous studies are indicated within brackets ([ ]) and the numbers correspond to those in the Supplementary References list.

File Name: Supplementary Data 2

Description: Genotypes of 45 polymorphic inversions in 551 individuals from seven different populations. The Family and Relationship columns summarize the information of the 30 CEU and 30 YRI known trios, plus 10 additional individuals with cryptic relationships identified from sequence data. Individuals in common with those included in the 1000 Genome Project (1000GP) Phase 1 (Ph1) and Phase 3 (Ph3) are also indicated. For each inversion, the chromosome in which it is located is shown in parenthesis. The genotypes described here correspond to version 4.8, which includes the latest information from the PCR/iPCR validations, and is the one used throughout the work. The only exceptions are the inversion recurrence analysis from haplotypes and the functional analysis that use the previous version 4.7. The two datasets differ by the addition of 10 new genotypes and 11 genotype corrections, which affect a total of 10 inversions. ND, not determined.

File Name: Supplementary Data 3

Description: Conservative estimate of number and distribution of recurrence events in human inversions mediated by non-allelic homologous recombination (NAHR). Each recurrence event was located in one or more population groups according to the individuals that unequivocally support it. Each individual event is named by a letter (A, B, C, D, etc.) and the direction is shown (from O1 to O2 as 1►2, for example). The number of affected chromosomes or individuals supporting recurrence was determined only from phased haplotypes of 1000GP Phase 1 data, excluding GIH individuals. NA, not analyzed due to lack of power to detect possible recurrence events (no haplotype clusters differentiated by at least 3 changes in more than 2 kb).

File Name: Supplementary Data 4

Description: Summary of Chr. Y haplogroups of males genotyped for the HsInv0832 inversion. Consensus haplogroup was obtained from different published studies, which are indicated in the

References column within brackets ([ ]) with the same number as in the Supplementary References list.

File Name: Supplementary Data 5

Description: Experimental genotyping of human inversions in chimpanzees and gorillas. Inversions HsInv0055, HsInv00374 and HsInv1051 are not included in the table because they could not be genotyped in either species with the human assays and it was not possible to design new ape-specific assays. The same happens for other inversions with missing information in one species. The breakpoints and assays analyzed include 40 inversions in chimpanzee, with 26 by one and 14 by both breakpoints (16 MLPA, 5 iMLPA, 3 PCR, 11 iPCR, 1 MLPA+PCR, and 4 iMLPA+iPCR) and 41 inversions in gorilla, with 30 by one and 11 by both breakpoints (15 MLPA, 4 iMLPA, 6 PCR, 11 iPCR, 1 MLPA+PCR, and 4 iMLPA+iPCR). Genotypes of parent-child trios and father-son pairs in chimpanzees and gorillas were consistent with expected genetic transmission in each inversion. The number of independent alleles analyzed for autosomal and chr. X inversions is, respectively, 37 and 28 in chimpanzees and 13 and 9 in gorillas. For HsInv0832 in chr. Y there are 9 independent alleles in chimpanzees and 4 in gorillas. ND, not determined.

File Name: Supplementary Data 6

Description: Age estimation and orientation of polymorphic inversion regions in non-human primates and ancient hominin genomes. Ancestral orientation was estimated from the consensus of experimental results and genome analysis for chimpanzee (panTro5), gorilla (gorGor5), orangutan (ponAbe2) and rhesus macaque (rheMac8). When the inversion was polymorphic in chimpanzees or gorillas (*O1+O2*), it was considered recurrent. For NAHR inversions, no final ancestral orientation was defined (ND) if there was not experimental support in at least one species (HsInv0055, HsInv0278 and HsInv0374), and in case of inconsistencies the orientation validated experimentally is given. Orientation of inversions without IRs in ancient hominin genomes was determined by mapping the sequencing reads in the breakpoint regions and a particular orientation was considered to be reliably identified if it was supported by at least two reads from any breakpoint (with the number of reads supporting each orientation indicated in parenthesis). Ancient genome references are indicated within brackets ([ ]) and the numbers correspond to those in the Supplementary References list. Results indicating ancient recurrence or polymorphism are shown in boldface. Inversion age was estimated with a constant (10^{-9} changes per bp per year) and two different local substitution rates based on divergence with chimpanzee and gorilla and the 95% confidence interval (CI) from bootstrap sampling is indicated in parentheses. ND, not determined. NA, not applicable.

File Name: Supplementary Data 7

Description: Summary of results of selection tests on inversions and inversion regions. Evidence of positive (F_{ST} and LSFS-Positive) or balancing (LSFS-Balancing, NCD1 and NCD2) selection is classified as strong (green) or weak (yellow) according to the following criteria: for F_{ST}, strong and weak mean *P* value < 0.01 and *P* value < 0.05, respectively; for LSFS-Positive and LSFS-Balancing, strong means conservative global (GLB) *P* value < 0.01 and weak means some population *P* value < 0.05 and a global conservative or approximate *P* value < 0.1; and for NCD1 and NCD2, strong and weak mean three or more populations with *P* value < 0.01 and *P* value < 0.05, respectively. Populations interpreted to be leading the F_{ST} population differentiation signal are indicated. For NCD1 and NCD2 statistics, the target frequency is the closest to the global inversion MAF between 0.3, 0.4 or 0.5.

File Name: Supplementary Data 8

Description: Results from the gene-expression analysis of inversions in the lymphoblastoid cell lines RNA-Seq data from the GEUVADIS project. Search of eQTL effects of 42 inversions in *cis* (± 1 Mb) was done at the level of genes and transcripts and both considering only the 173 experimentally-genotyped individuals (experimental) and the 445 Geuvadis individuals in the 34 inversions in which the genotypes could be reliably imputed (imputed). Gene and transcript information was obtained from GENCODE version 26 and the distance was calculated between the gene and the closest breakpoint of the inversion. For inversions located within an intron the distance is 0. Only inversion eQTLs with FDR < 0.05 are listed and it is indicated if they are lead eQTLs compared to other variants or not.

File Name: Supplementary Data 9

Description: Inversion effects on gene expression across different tissues from the GTEx project. Inversion effects were estimated through variants showing the highest linkage disequilibrium (LD) with the inversion, corresponding to either tag SNPs ($r^2 \geq 0.8$) or linked SNPs with a more moderate association for recurrent inversions ($0.6 \leq r^2 < 0.8$), that have been reported as *cis*-eQTLs in different tissues in GTEx Analysis Release v7. Lead eQTLs correspond to those in which the inversion shows the highest LD with the first or second lead eQTL for the expression variation of the gene in the GTEx analysis.

File Name: Supplementary Data 10

Description: Probes used in direct (MLPA) and inverse (iMLPA) MLPA experiments to genotype 41 human inversions. MLPA (68) and iMLPA (87) probes were designed using the program Proseek[87] and manually modified to hybridize around the inversion breakpoints (MLPA) or the self-ligated restriction-enzyme target site (iMLPA) sequences, taking into account the usual MLPA-probe recommendations[30] and that no common SNPs were close to the ligation ends of the probes. The common primer sequence is shown in black, the stuffer in blue and the specific probe sequence in red. Probe conc. correspond to concentrations in the probe mixes for MLPA or iMLPA, not in the final hybridization reaction.

File Name: Supplementary Data 11

Description: Primers used in inversion genotyping experiments.

File Name: Supplementary Data 12

Description: List of SNPs and small indels in high linkage disequilibrium (LD) ($r^2 \geq 0.8$) with the studied inversions. LD was estimated separately per population (LWK, YRI, CHB, JPT, CEU, TSI, GIH), population group (AFR, EAS, EUR) or globally (GLB) for both the 1000 Genomes Project (1000GP) Phase 3 and HapMap project data. Overall, LD results from 1000GP and HapMap are highly concordant, with 27/29 of the 1000GP perfect global tag SNPs tested in HapMap having also perfect LD with the inversion. In addition, HapMap data adds three perfect tag SNPs in the list that are either in high LD or were not reported in 1000GP. NA, inversion not polymorphic in the population or SNP not found in 1000GP or HapMap.

File Name: Supplementary Data 13

Description: Breakpoint library used to identify sequence reads from the two orientations of 19 inversions with the BreakSeq algorithm. Sequences included within the inverted region are indicated in red, and blue sequences denote those nucleotides present in one allele and absent in the other.
